# Americans preferred Syrian refugees who are female, English-speaking, and Christian on the eve of Donald Trump’s election

**DOI:** 10.1371/journal.pone.0222504

**Published:** 2019-10-10

**Authors:** Claire L. Adida, Adeline Lo, Melina R. Platas

**Affiliations:** 1 Department of Political Science, University of California, San Diego, La Jolla, CA, United States of America; 2 Department of Political Science, University of Wisconsin-Madison, Madison, WI, United States of America; 3 Division of Social Science, New York University Abu Dhabi, Abu Dhabi, United Arab Emirates; University of California-Irvine, UNITED STATES

## Abstract

What types of refugees do Americans prefer for admission into the United States? Scholars have explored the immigrant characteristics that appeal to Americans and the characteristics that Europeans prioritize in asylum-seekers, but we currently do not know which refugee characteristics Americans prefer. We conduct a conjoint experiment on a representative sample of 1800 US adults, manipulating refugee attributes in pairs of Syrian refugee profiles, and ask respondents to rate each refugee’s appeal. Our focus on Syrian refugees in a 2016 survey experiment allows us to speak to the concurrent refugee crisis on the eve of a polarizing election, while also identifying religious discrimination, holding constant the refugee’s national origin. We find that Americans prefer Syrian refugees who are female, high-skilled, English-speaking, and Christian, suggesting they prioritize refugee integration into the U.S. labor and cultural markets. We find that the preference for female refugees is not driven by the desire to exclude Muslim male refugees, casting doubt that American preferences at the time were motivated by security concerns. Finally, we find that anti-Muslim bias in refugee preferences varies in magnitude across key subgroups, though it prevails across all sample demographics.

## Introduction

What types of refugees do Americans prefer to admit into the United States? We know that Americans prefer high-skilled English-speaking immigrants [1], and that Europeans prioritize asylum-seekers with higher employability and greater humanitarian need [2]. We also know that anti-Muslim bias pervades American politics [3] as well as public preferences for immigrants and asylum-seekers [1, 2, 4]. Yet our knowledge of American preferences towards refugees, a particularly vulnerable population, and in a country which until recently accepted the largest number of resettled refugees annually, can be broadened. In light of record-high forced displacement globally, policy changes regarding migration and refugee resettlement under the Trump administration, and the politicization of the admissions process of refugees and asylum-seekers in the United States, it is worth asking what kinds of refugees the American public prefers.

The literature on immigrant exclusion is theoretically and empirically rich, indicating that immigrant exclusion is driven primarily by sociotropic cultural threats, with less evidence for individual economic threat (e.g., [1, 5], though see [6, 7]). We lack a similarly clear understanding of what drives public attitudes toward *refugees*. There are reasons to believe that public opinion toward refugees might be qualitatively different than that toward immigrants. Refugee status is a legal determination, and by definition, refugees migrate due to fear of persecution. The US Citizenship and Immigration Services agency defines a refugee as someone who, among other criteria, is of special humanitarian concern to the United States, and demonstrates that they were persecuted or fear persecution due to race, religion, nationality, political opinion, or membership in a particular social group [8]. Meanwhile, the term “immigrant”, though technically encompassing those who migrate for any reason, colloquially tends to refer to economic migrants, who leave their home country for economic reasons.

There are at least two ways in which the public might perceive refugees as different from (economic) immigrants. On the one hand, the public may view refugees as vulnerable individuals who face persecution in the country of origin and whose decision to migrate is involuntary. Indeed, humanitarian concerns are an important factor driving European attitudes toward asylum-seekers [2]. If so, we might expect the public to prefer those individuals they deem as particularly vulnerable, for example, women and children.

On the other hand, the public may believe that refugees pose a greater security risk than immigrants either because refugees come from a conflict zone or because the public misunderstands the refugee screening process as more lenient than it is in fact. A 2016 survey found that a majority of respondents in eight out of ten European countries believed that refugees would increase the likelihood of terrorism [9]. Additionally, a significant portion of refugees resettled in the United States today originate from Muslim-majority countries such as Syria, Iraq, and Somalia [10], such that American Islamophobia [11] could shape public beliefs about refugees as a security threat. Therefore, security concerns may drive preferences in refugee profiles, such that the public screens profiles based on attributes or combinations of attributes they deem likely to be associated with greater security risk, such as young men [12]. Indeed, security concerns motivated President Trump’s executive order banning entry into the United States of aliens from certain Muslim-majority countries.

Drawing on a nationally representative sample of 1,800 American citizens, and on more than 5,000 conjoint experiments which we administered in October 2016, we identify Americans’ preferred refugee profile on the eve of a a polarizing presidential election. Our empirical strategy focuses on American preferences among refugees from Syria for both substantive and methodological reasons. As of mid-2017, nearly one-third of all registered refugees were Syrian—close to 6 million—making the Syrian case a substantively important one [13]. At the same time, Syria is home to both Muslims and Christians, allowing us to identify, if it exists, anti-Muslim bias while holding constant national origin.

Our findings indicate American preferences for refugees are broadly in line with preferences for immigrants, as identified in existing work [1], though we also find evidence that Americans privilege women, and that this is not driven by a targeted rejection of Muslim male refugees, casting doubt that Americans were motivated by security concerns at the time.

Specifically, our findings show that the American public prefers Syrian refugees who are female, high-skilled, English speakers, and Christian. The most consistent and substantive determinant is religion: Muslim profiles rate on average 0.5 points lower than do Christian profiles, a substantive difference for a scale that runs from 1 (the respondent believes the United States should absolutely not admit the refugee) to 7 (the respondent believes the United States should definitely admit the refugee).

Further, we find that the anti-Muslim bias in Syrian refugee preferences in 2016—while manifest across all subgroups we measure in our sample—is significantly lower for Democrats (e.g., [14]), for non-whites, and for non-Christians. Islamophobia motivated American refugee preferences in 2016, though not equally for all respondent-types.

Understanding the preferences, and biases, of the American public with respect to refugees is important both theoretically and with respect to policy. Immigration, broadly speaking, is an issue that has long held “flash potential”—the potential for large-scale mobilization of the public [15]. While historically immigration debates in the U.S. have largely concerned economic migrants, in recent years the focus has turned more toward migrants fleeing instability and conflict, particularly from the Middle East as well as more recently from Latin America. Given the flash potential of this issue for electoral politics, it is important to understand specifically how the American public views not just immigrants in general, but refugees in particular.

The number of refugees resettled annually in the U.S. has been dramatically decreased since the survey was fielded, and resettlement of Syrians and Muslims in particular has dropped precipitously, both in absolute and percentage terms, despite the large number of refugees fleeing Syria and Muslim-majority countries. Public attitudes—and especially partisan attitudes—toward refugees may play an important role in shaping legislators’ behavior [16], with implications for thousands of those seeking refuge from conflict. This paper contributes to a recent but vibrant literature that examines attitudes and behavior toward refugees and asylum seekers around the world, as well as anti-Muslim sentiment among the American public [2, 3, 14, 17–20].

## Materials and methods

We test which factors drive American preferences toward Syrian refugees with a conjoint experiment conducted in October-November 2016, just prior to the 2016 U.S. elections. We fielded the survey during this period precisely because the refugee crisis had become such a dominant issue in the public sphere, and yet at the time there was little research on attitudes toward refugees in the U.S. beyond public opinion surveys. While we could not have known the outcome of the presidential election, in retrospect, the timing of our survey serves as a unique lens into the attitudes of the American public before the implementation of a set of more extreme exclusionary policies with respect to refugees under the Trump administration. These included Executive Order 13769, which banned the entry into the U.S. of citizens from a set of Muslim-majority countries and suspended the entry of Syrian refugees indefinitely; additionally, the Trump administration revised the annual refugee cap downward from 110,000 during President Obama’s final year to 50,000 in 2017, 45,000 in 2018, and 30,000 in 2019.

Relying on YouGov, we procured a nationally-representative sample of 1,800 American adult citizens, and fielded 5,400 conjoints in an online survey, with a total of 10,800 refugee profiles. YouGov provides a representative sample of American citizens via matching on gender, age, race, education, party identification, ideology and political interest with the 2010 American Community Survey, the November 2010 Current Population Survey, and the 2007 Pew Religious Life Survey. A detailed description of YouGov’s sampling strategy is available in the Supplementary Information. We note that relying on online surveying has its limitations, especially as there is no comprehensive way to ensure virtually every American citizen has a chance of being selected. However, in a recent Pew study evaluating the accuracy and biases of online surveys, YouGov (known as ‘Vendor I’ in the report) was found to consistently outperform the other nine surveying vendors [21].

Table 1 provides summary statistics for our sample. Our sample is 55% female, 79% white, 9% Black, and 7% Hispanic. Thirty-nine percent of respondents are high school graduates, while more than half have attended college (2 or 4 years). Nearly half of respondents are employed, either full or part time, 19% are retired, and 6% are unemployed. The mean respondent age is 48 years. Approximately one-third identifies as Democrat, one-third as Independent, and one-quarter as Republican. The median family income is $50,000-59,000. Fourteen percent of sampled respondents are first-generation immigrants (defined here as US-born respondents of at least one foreign-born parent), and 20% are second-generation immigrants.

**Table 1:** Summary statistics of respondents.

	Mean	SD	Min	Max
Age	48.294	16.690	18	95
Female	0.546	0.498	0	1
US-born	0.944	0.230	0	1
First generation immigrant	0.141	0.348	0	1
Second generation immigrant	0.199	0.400	0	1
Ethnocentrism	2.077	0.390	1	3
*Race*				
White	0.790	0.408	0	1
Black	0.086	0.280	0	1
Hispanic	0.069	0.253	0	1
Mixed-Race	0.021	0.142	0	1
*Religion*				
Protestant	0.343	0.475	0	1
Catholic	0.201	0.401	0	1
Muslim	0.008	0.088	0	1
Jewish	0.021	0.144	0	1
*Education*				
High school	0.386	0.487	0	1
College	0.527	0.500	0	1
Post-graduate	0.087	0.281	0	1
*Party*				
Democrat	0.348	0.477	0	1
Republican	0.243	0.429	0	1
Independent	0.338	0.473	0	1
*N*	1800			

**Fig 1:**
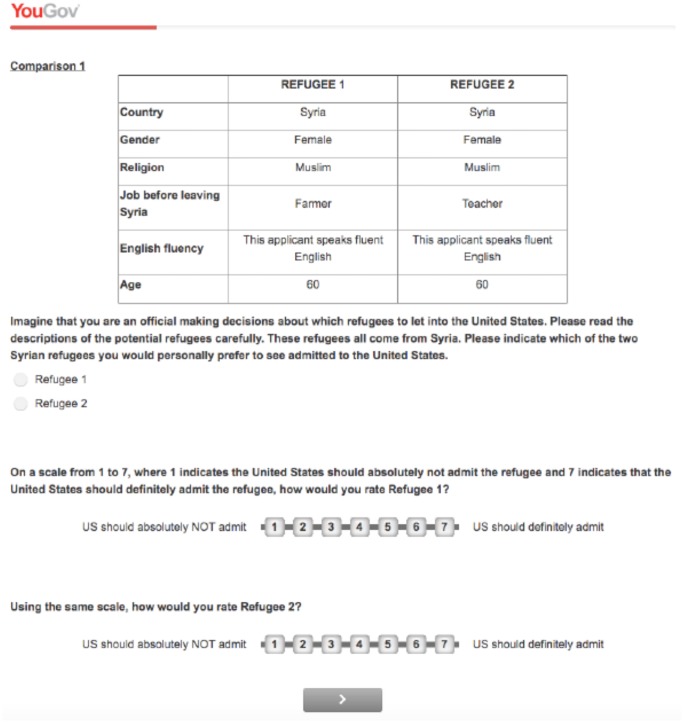
Screenshot of the conjoint experiment.

Conjoint analysis is a common methodological approach in marketing, but it was first introduced to political science by Hainmueller, Hopkins and Yamamoto [22]. Since then, conjoint design has been used to isolate the stated preferences of Americans for various immigrant characteristics [1], and of Europeans for various asylum-seeker characteristics [2]. Similarly, we rely on this design to isolate Americans’ stated preferences toward refugee characteristics. The dimensions describing the refugees are listed below, and their values were randomly assigned (see Fig 1 for a screenshot). Respondents were then asked to rate each refugee on a scale from 1 (the US should absolutely not admit the refugee) to 7 (the US should definitely admit the refugee), and then to choose one for admission into the US:

- Country: Syria (constant)
- Gender: Male/Female
- Religion: Christian/Muslim
- Previous occupation: Farmer/Teacher/Doctor
- English fluency: Fluent/Broken/Interpreter
- Age: 20/40/60

Relying on the conjoint design allows us to reach three key objectives. First, the randomization of refugee characteristics on a set of dimensions allows us to identify the independent effect of each refugee characteristic while also making it easier to compare each source of discrimination. It is possible, for example, to identify anti-Muslim bias through conjoint analysis because we provide profiles that are randomly assigned a Muslim versus a Christian religion. Therefore, holding all other dimensions in the conjoint constant, we can evaluate the effect of being Muslim (versus being Christian) on a respondent’s refugee ratings. The conjoint design also makes it possible to easily compare respondent preferences based on refugee religion to respondent preferences based on refugee gender or English fluency, etc… in other words, it offers an intuitive way to compare the size of respondent biases across each conjoint dimension. Second, the conjoint method allows us to present a holistic comparison of refugees in a format that is easy to read and understand for the user. Third, this method has been widely used for understanding public opinion toward immigrants and has been shown to perform better than vignette experiments in approximating a behavioral benchmark [23].

As with any design choice, the use of conjoint also presents limitations. First, we can only speak to respondents’ stated preferences over refugee characteristics that we, as researchers, chose to include in the design. Here, our choice of refugee-characteristics was guided both by the existing literature and common refugee narratives in the media. Specifically, our conjoint design focuses on characteristics—religion, gender, skill-level, English fluency, and age—previously shown to be salient for public opinion toward immigrants. In 2016, for example, [2] showed that Europeans prefer asylum-seekers with greater employability (higher-skilled) and who are Christian rather than Muslim. Prior to that, [1] found that Americans prefer immigrants with higher levels of education, with high-skilled jobs, and with greater English fluency. They further found that Iraqi immigrants were penalized, though without elucidating the source of this discrimination. Our choice of conjoint dimensions therefore reflects findings from the prior literature that employability and cultural difference shape public opinion toward migrants. Furthermore, it reflects common narratives of refugees as an economic [24] and a cultural [25] threat to the country. Our conclusions speak only to the relative importance of refugee dimensions we chose as researchers. We cannot speak to refugee characteristics we did not include in our research design. We were careful not to overload the conjoint design with too many profile characteristics in order to avoid possible respondent fatigue or reliance on information shortcuts.

Second, the conjoint design does not isolate attribute-preferences compared to null baselines—profiles that simply do not embody such attributes. For example, we cannot say anything about absolute preferences for female profiles; we can only estimate preferences for female profiles compared against male profiles, holding all other profile characteristics constant. The conjoint design therefore does not allow us to make claims about the absolute intensity of preference. But including null baselines would violate information equivalence: respondents may infer *different* levels of the missing attribute based on surrounding information of all other attributes in the profile [26], or even based on their own biases. If respondents assume that all refugees are Muslim, for example, they may infer that a baseline refugee profile (one that mentions no religion attribute) is Muslim, making it difficult to identify a Muslim effect that exists.

Finally, our conjoint presented survey respondents with three pairs of randomized refugee profiles—presented sequentially—and asked them, after each pair, to imagine that they are an official deciding which refugee to allow into the country for resettlement. Yet ordinary American citizens are never in a position to evaluate refugees in the real world, as they might in Switzerland through referenda on immigrant naturalization decisions [27]. Still, this type of thought exercise is similar to that conducted by [22] and [1] asking American respondents to evaluate immigrant profiles. It is analytically useful for the purpose of isolating refugee characteristics that are appealing to the American public. It is also relevant to the extent that political elites respond to public opinion (e.g., [28]).

The nature of the conjoint experiment involves the randomization of each attribute, such that the probability that each attribute appears in a given profile is orthogonal to that of all other attributes. This makes estimating the treatment effect of any given attribute on the outcome a straight-forward process. Our estimand of interest is the average marginal component effect (AMCE) [22]. This is the average difference in refugee rating when comparing two attribute values and averaging over all possible combinations of the other profile attributes. The statistical model used is a regression of the rating outcome on indicator variables for levels of each attribute.

We focus on the ratings outcome rather than the forced choice outcome for two reasons. First, the ratings outcome allows the respondent to reject both profiles in a pair and therefore facilitates a more intuitive interpretation of results. Second, the ratings outcome allows us to address the fact that respondents may vary in their intensity of preferences; by contrast, the forced choice outcome assigns greater weight to respondents with more intense preferences over attributes, which can lead to inaccurate out-of-sample predictions. We note that our results hold when we rely on the forced choice outcome rather than the refugee rating.

Additionally, because each respondent views three pairs of profiles, and individual respondents may evaluate them in correlated ways, we cluster the standard errors at the respondent level. Finally, we estimate and present unweighted results, but in robustness checks we verify that these hold when utilizing weights. Weighted results can be found in the Supplementary Information section.

## Results and discussion

What types of refugees do Americans prefer? Our conjoint analysis reveals two key findings. First, American respondents prefer high-skilled female Christian Syrian refugees who speak fluent English. And second, the Muslim penalty, which prevails among a large cross-section of respondents, is significantly more pronounced for self-identified Republican, white, and Christian respondents. Our conjoint design was pre-registered with the Evidence on Governance and Politics site (egap.org/registration/2235). This study was part of a larger survey experiment testing the effectiveness of messages designed to increase refugee inclusion. In the below analysis, only the anti-Muslim bias was preregistered. All other tests and results are pattern discoveries.

**Fig 2:**
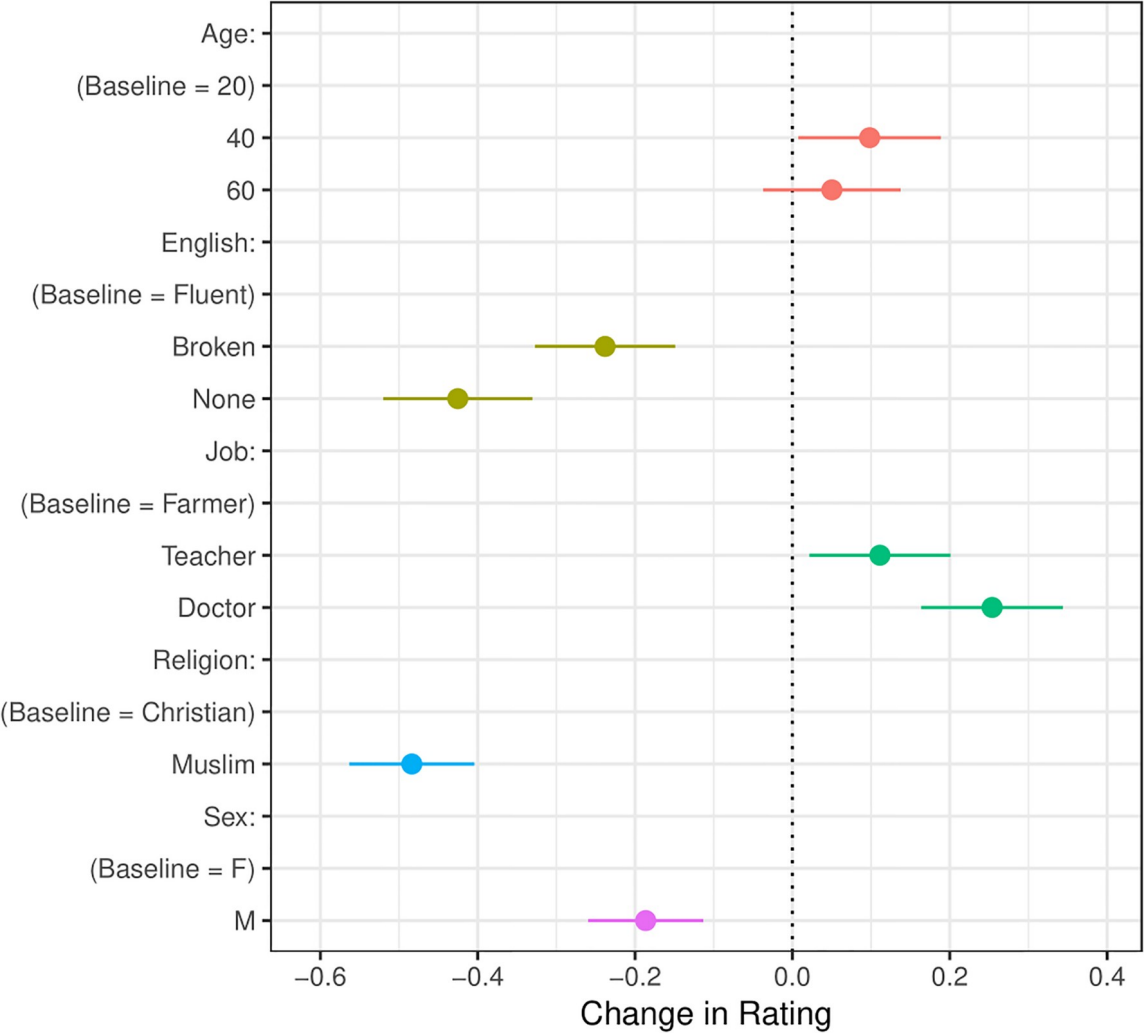
Average marginal components effect plot. Confidence intervals are at 95%.

**Fig 3:**
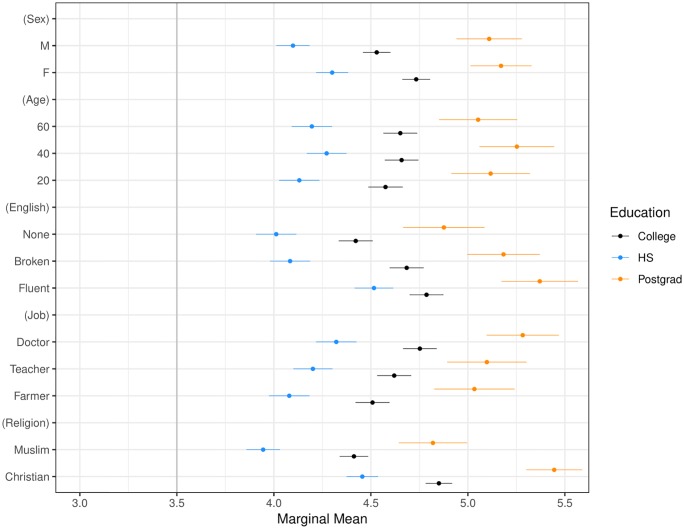
Marginal means plot by respondent education. Education categories are respondents who have high school, college, and post-graduate degrees. Confidence intervals are at 95%.

Fig 2 illustrates our main result: our respondents prefer middle-aged, high-skilled, female Christian Syrian refugees who speak fluent English (all confidence intervals throughout all our Figures are at the 95% level). The effects are substantively strongest for language-fluency and religion, a result that echoes the cultural-threat literature on immigrant exclusion. While this pattern persists across subgroup analyses by party, race and respondent religion, we note that white Christian respondents who identify as Republican assign lower refugee ratings across the board compared to non-white, non-Christian Independents or Democrats. Additionally, we find no significant heterogenous treatment effect by respondent skill-level, as proxied by education (see Fig 3). In other words, all respondents prefer high-skilled refugees, consistent with findings in the literature on immigrant exclusion that individual economic competition is a weaker predictor of immigrant exclusion than are cultural or sociotropic concerns.

To be sure, this test is imperfect: we proxy the respondent’s skill level with a measure of educational attainment, rather than with a direct measure of skill. Scholars have previously argued that imperfect proxies for skill might yield misleading claims [7, 29]. As an alternative test, we examine whether refugee preferences are significantly different for respondents who are not in the labor force, as respondents in the labor force are more likely to be sensitive to refugees’ economic potential than respondents outside the labor force. We find that they are not.

The large and significant negative effect for Muslim profiles is important, albeit unsurprising given the anti-Muslim discrimination previously documented in the immigrant exclusion literature (e.g., [1, 2, 4]) and the degree of Islamophobia identified in American society [11]. At the same time, we are the first to document this anti-Muslim bias toward Syrian refugees among a representative sample of American adults. Additionally, we see a large and significant positive effect for female profiles.

**Fig 4:**
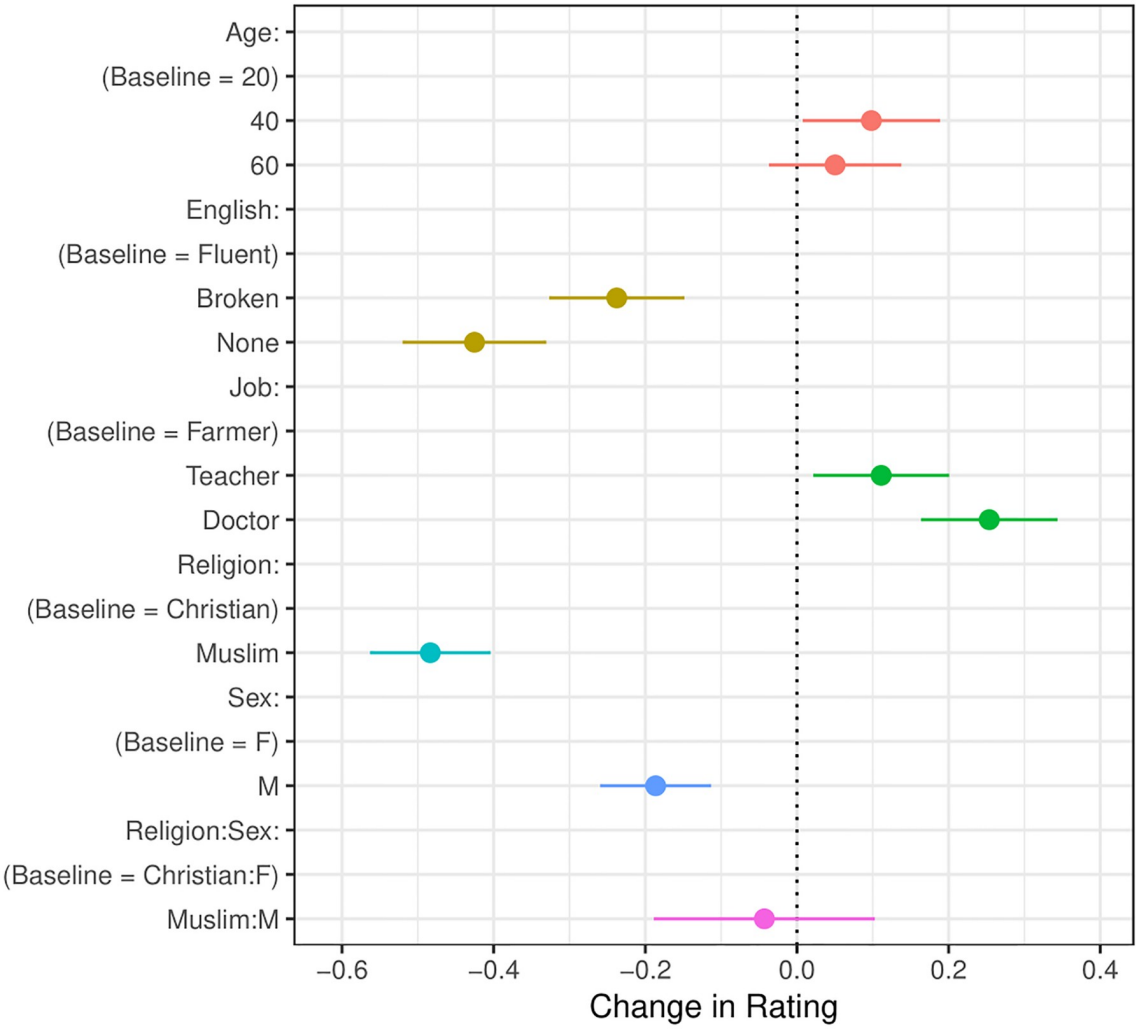
Average marginal components effect with interaction between refugee religion and refugee gender. Confidence intervals are at 95%.

**Fig 5:**
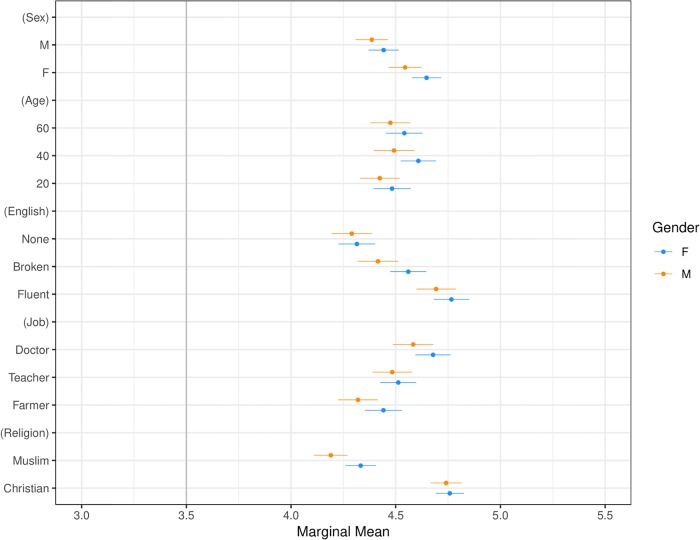
Marginal means plot by respondent gender. “F” are female respondents and “M” indicate male respondents. Confidence intervals are at 95%.

Taken together, these results raise a question: is anti-Muslim discrimination driven by a perceived security threat? That is, do Americans exclude Muslim male refugees specifically due to a higher perceived security threat with this profile? Further analysis indicates that respondent preferences for female profiles are **not** driven by a desire to exclude Muslim male profiles, specifically: the interaction effect between the gender and religion of the profile, while in the expected direction (it is negative for Muslim male profiles), is not statistically significant (see Fig 4). We further investigate whether any respondent-type is more likely to specifically exclude Muslim male profiles and find consistently null results. In sum, our respondents prefer female over male profiles, and this is driven neither by the desire to specifically exclude Muslim male profiles (Fig 5), nor by certain respondent-types who may be more sensitive to security concerns. Public opinion polls suggest Republican voters are more concerned with national security issues than are Democratic or Independent voters (e.g., [30]). These results are preliminary, but question the claim that, on the eve of the 2016 presidential election, Americans perceived a security threat from Syrian refugees.

**Fig 6:**
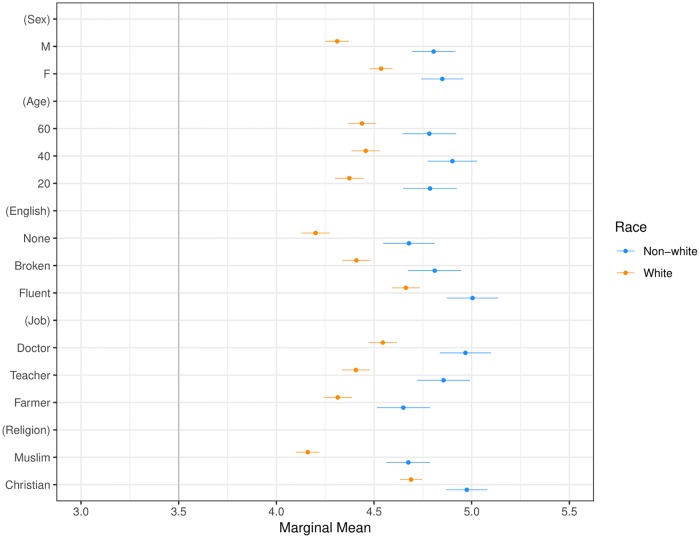
Marginal means plot by respondent race. Categories include White and non-White respondents. Confidence intervals are at 95%.

**Fig 7:**
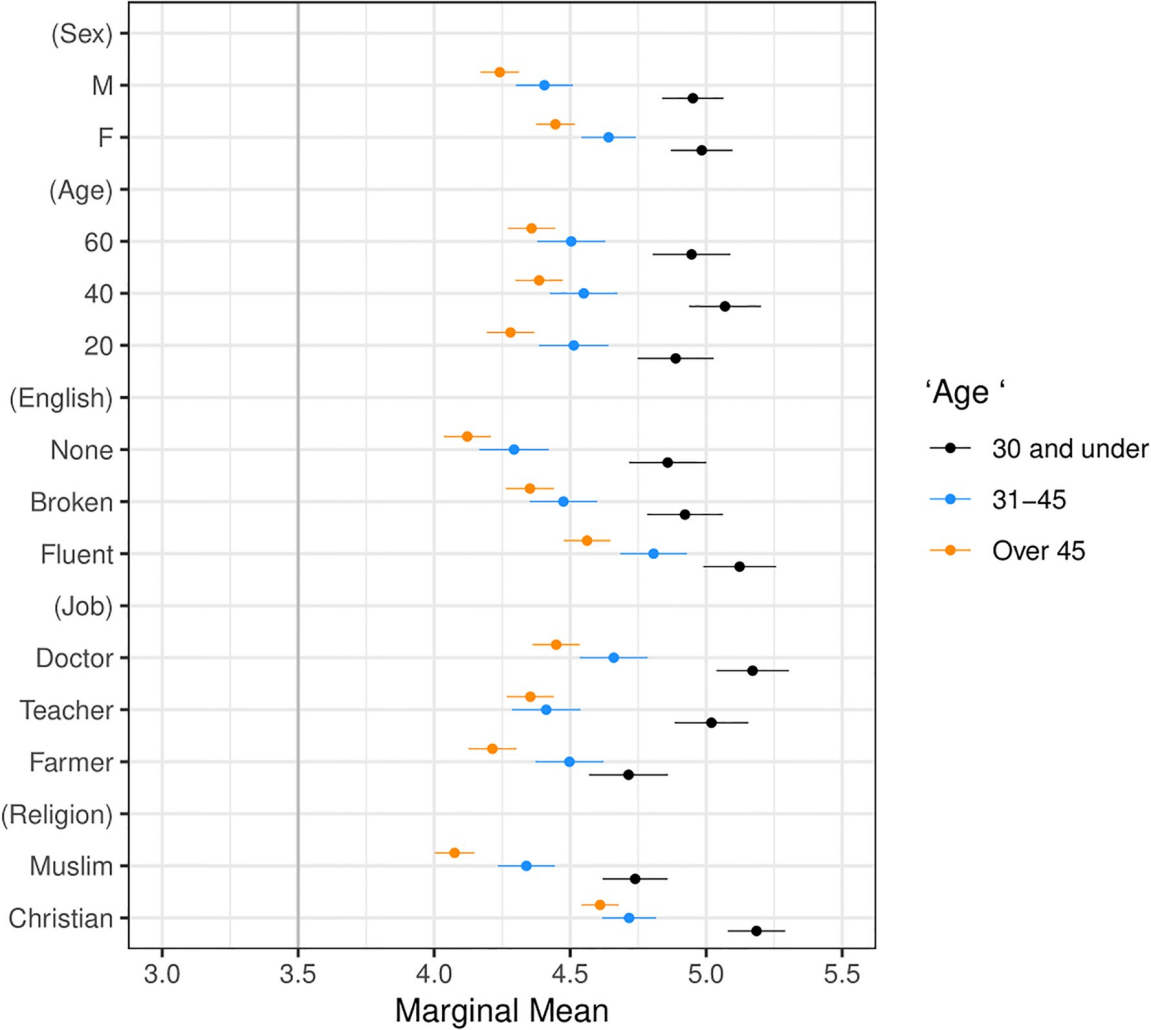
Marginal means plot by respondent age. Categories are age 30 and under, ages 31-45, and over 45. Confidence intervals are at 95%.

**Fig 8:**
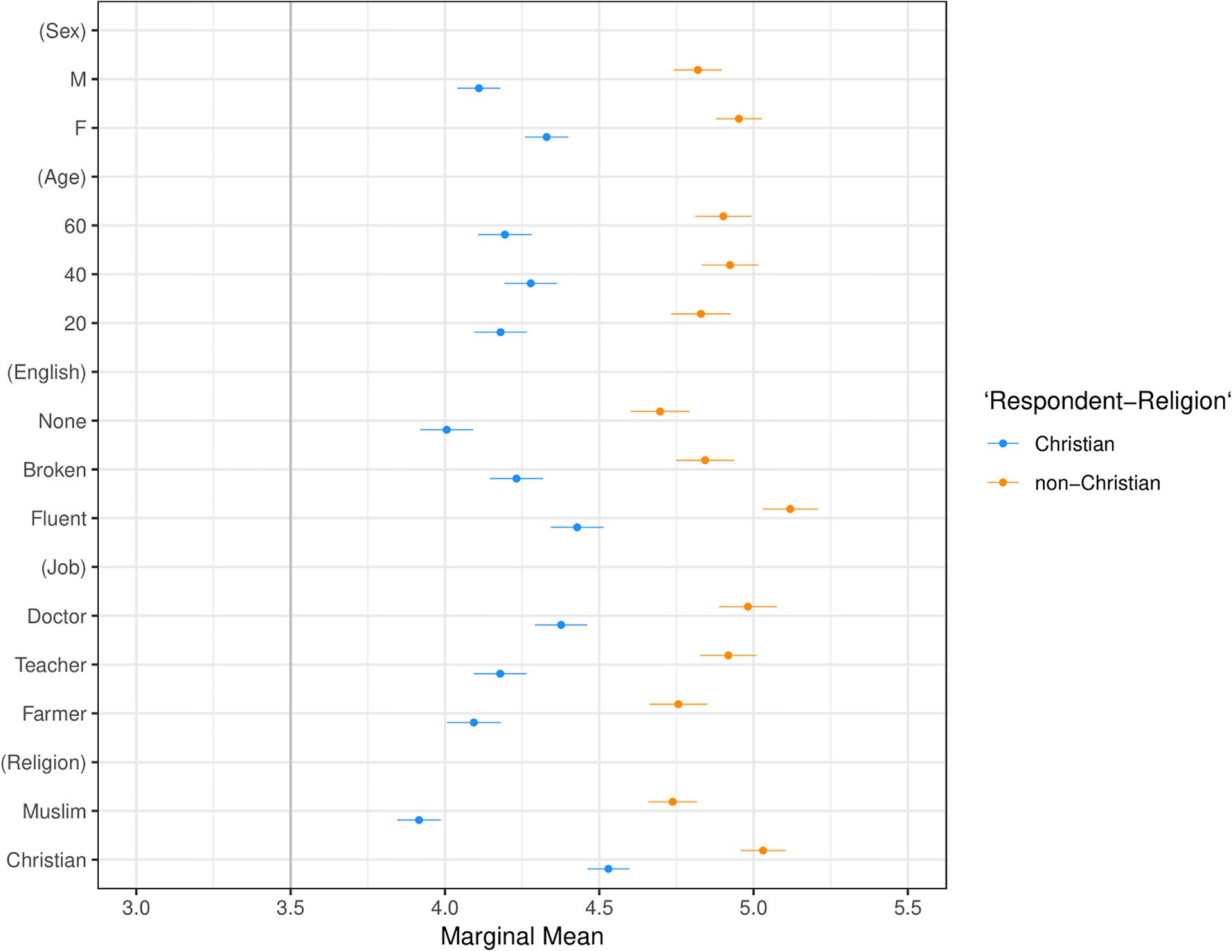
Marginal means plot by respondent religion. Categories are Christian and non-Christian. Confidence intervals are at 95%.

**Fig 9:**
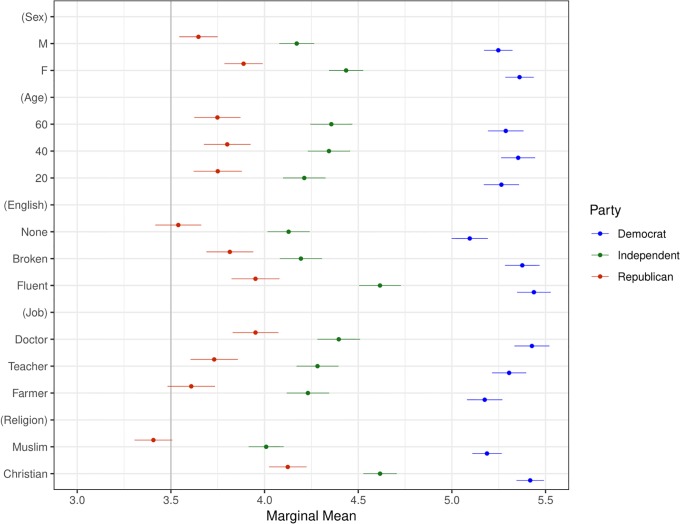
Marginal means plot by respondent political party. Categories are Democrat, Independent, and Republican. Confidence intervals are at 95%.

**Fig 10:**
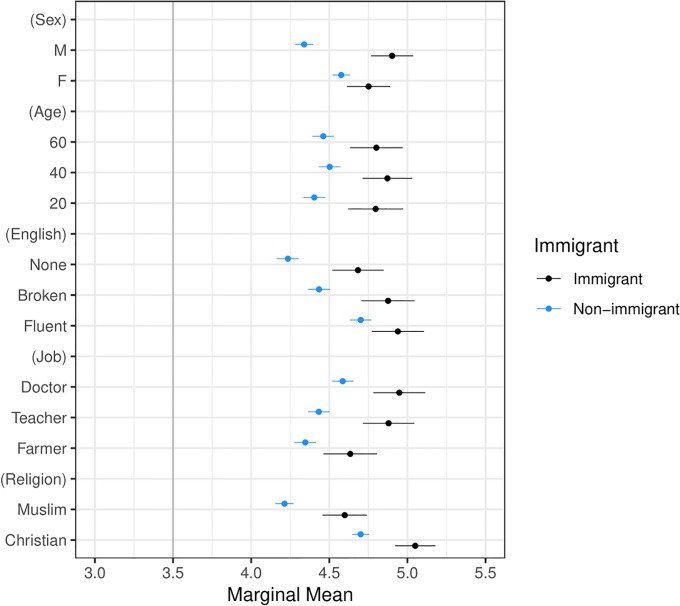
Marginal means plot by respondent immigration history. Respondents in the “Non-immigrant” category are a) born in the U.S. and have parents who are born in the U.S. but have at least one grandparent who is an immigrant or b) born in the U.S. and have parents and grandparents who are also born in the U.S. Respondents in the “Immigrant” category are either a) self-identified immigrants to the U.S. who are naturalized citizens or b) born in the U.S. but have at least one parent who is an immigrant to the U.S. Our findings are robust to different definitions of these two groups (see S8 and S9 Figs in the Supplementary Information).

Our second main result, presented in Figs 3 and 5–9 shows that the Muslim penalty, which prevails among a large cross-section of respondents, is significantly more pronounced for self-identified Republican, white, and Christian respondents. Indeed, while the preference for high-skilled, English-speaking, Christian profiles is widespread in our sample, the anti-Muslim bias is moderated by respondent characteristics, such as race, religion and political party affiliation. For each subgroup we calculate marginal mean values for profile attributes to characterize differences in preferences between subgroups in a manner robust to reference category choice [31]. We find that non-White respondents exhibit a significantly smaller anti-Muslim bias than do White respondents (*p* < 0.05, see Fig 6). Likewise, non-Christian respondents exhibit nearly half the level of anti-Muslim bias than do their Christian counterparts (*p* < 0.001, see Fig 8). Finally, respondents who self-identify as Democrat, Independent or Republican all prefer Christian refugee profiles to Muslim profiles on average, but the magnitude of this bias differs by party. Respondents who self-identify as Democrats demonstrate significantly less anti-Muslim bias than respondents who self-identify as Independents and Republicans (*p* < 0.001, see Fig 9). We also analyze whether refugee preferences differ for respondents who are themselves closer to the immigrant experience. Recent work has shown that individuals who themselves share a history of forced migration exhibit greater inclusion of refugees [32]. We find that respondents who were immigrants themselves, or are children of at least one immigrant parent, are more likely to give higher ratings compared to respondents whose immigration experience is more distant (see Fig 10). Furthermore, such respondents are less discerning across attribute levels.

## Conclusion

This paper improves our understanding of American public attitudes toward refugees, explicitly testing and capturing religious discrimination in refugee preferences in the US. Our results complement those of [14], who finds that self-identified Republicans and conservatives are less likely to support the admission of Syrian refugees. They also suggest that Americans assess refugees in similar ways in which they assess immigrants more broadly [1], and that Americans and Europeans are similarly motivated by anti-Muslim bias [2]. But our results also call for more investigation into whether or not separate concerns—namely humanitarian and security concerns—motivate the American public’s preferences for refugees. Our respondents’ large and significant preference for female refugees over male refugees—a preference not motivated by the desire to exclude Muslim male refugees specifically, suggests that vulnerability concerns might also matter in shaping American preferences for Syrian refugees, while security concerns may be less apparent. We note that adding a conjoint dimension about security threat is difficult for both research design and ethical reasons. Including an attribute on extent of security screening may lead respondents to falsely believe that some refugees do not undergo extensive security screening, while an attribute on the likelihood of committing a crime is both unrealistic and misleading as there is no way this information could be systematically compiled. Further, anyone thought to pose any sort of threat would not make it through the screening process in the first place. Future research should further this line of inquiry by explicitly testing the extent to which and the reasons why the American public might differentiate between refugees and immigrants.

Public attitudes toward refugees, and particularly partisan attitudes, likely shape the behavior of policymakers [16], with potentially dire consequences for those seeking refuge. Recent research has shown that anti-Muslim discrimination and divisive campaigns can cause Muslims in the U.S. to reduce their online visibility and retreat from public life [33]; additionally, anti-Muslim animosity contributes to online radicalization among Muslims in Western Europe [34]. The consequences of anti-Muslim sentiment such as that identified in this paper are therefore potentially significant.

Since the fielding of our survey in 2016, the issue of refugee resettlement and asylum-seeking has only intensified in public discourse. Though our survey was conducted at an arguably polarizing time—just prior to the U.S. presidential election—it is plausible that public opinion on refugees has become even more polarized, particularly along partisan lines. Indeed, the partisan gap on whether or not the U.S. has a responsibility to admit refugees grew in the year after President Trump took office. The percentage of Republicans who agreed that the U.S. does have a responsibility to accept refugees fell from 35 to 26 percent, while among Democrats, it increased from 71 to 74 percent [35]. Future research should continue to assess the implications of such polarization for refugee admissions, and evaluate strategies for reducing anti-Muslim sentiment, especially toward already vulnerable populations, such as refugees.

## Supporting information

S1 File Pre-analysis plan. (PDF)

S2 File Sampling information. (PDF)

S1 Fig Marginal means plot for subgroup analysis with weights by respondent party ID. Categories are Democrat, Independent, and Republican. Confidence intervals are at 95%.
(TIFF)

S2 Fig Marginal means plot for subgroup analysis with weights by respondent religion. Categories are Christian and non-Christian. Confidence intervals are at 95%.
(TIFF)

S3 Fig Marginal means plot for subgroup analysis with weights by respondent race. Categories include White and non-White respondents. Confidence intervals are at 95%.
(TIFF)

S4 Fig Marginal means plot for subgroup analysis with weights by respondent gender. “F” are female respondents and “M” indicate male respondents. Confidence intervals are at 95%.
(TIFF)

S5 Fig Marginal means plot for subgroup analysis with weights by respondent education. Education categories are respondents who have high school, college, and post-graduate degrees. Confidence intervals are at 95%.
(TIFF)

S6 Fig Marginal means plot for subgroup analysis with weights by respondent age. Categories are age 30 and under, ages 31-45, and over 45. Confidence intervals are at 95%.
(TIFF)

S7 Fig Marginal means plot for subgroup analysis with weights by respondent immigration history. Group 1 are respondents who are immigrants to the U.S. or have at least one parent who is an immigrant. Group 0 are respondents who are not immigrants, nor have parents who are immigrants, and either have no immigrants in their grandparents’ generation or at least one immigrant in their grandparents’ generation.
(TIFF)

S8 Fig Marginal means plot for subgroup analysis by respondent immigration history. Group 1 are respondents who are immigrants to the U.S., Group 2 respondents have at least one parent who is an immigrant, Group 3 has at least one grandparent who is an immigrant, and Group 4 has no immigrant history within their own, their parents’ and their grandparents’ generations.
(TIFF)

S9 Fig Marginal means plot for subgroup analysis with weights by respondent immigration history. Group 1 are respondents who are immigrants to the U.S., Group 2 respondents have at least one parent who is an immigrant, Group 3 has at least one grandparent who is an immigrant, and Group 4 has no immigrant history within their own, their parents’ and their grandparents’ generations.
(TIFF)
