# Supplementary material for: Americans preferred Syrian refugees who are female, English-speaking, and Christian on the eve of Donald Trump’s election
Source: PLoS One. 2019 Oct 10;14(10):e0222504. doi: 10.1371/journal.pone.0222504 (PMC6786519; doi:10.1371/journal.pone.0222504)
Supplement: S1 File — (PDF) [file pone.0222504.s001.pdf]

Determinants of refugee exclusion and its attenuation: The case of Syrian refugees to the U.S. Pre-analysis plan*

Claire L. Adida† Adeline Lo‡ Melina Platas Izama§

October 31, 2016

1 Research Question

A significant literature on immigrant exclusion has shown that individuals in industrialized democracies exclude immigrants when they feel threatened by them, either economically or culturally. But we know little about how to counter this exclusionary tendency. This project leverages the Syrian refugee crisis – one of the most significant humanitarian crises of our time – to test whether and how individuals might be pushed toward a more inclusionary approach. This study's contributions are four-fold:

1. The focus of the study is on how to move individuals toward *inclusion* rather than exclusion. This is a topic that has received very little attention, in particular in experimental political science.

2. The study will test two methods of persuasion – emotional (via an empathy prime) and rational (via a persuasion argument) – to assess whether and how individuals can be moved toward inclusion.

*This study is funded by an NSF RAPID Collaborative grant (SES-1503802) and by the UCSD Academic Senate. It was determined as exempt by the UCSD Institutional Review Board (Project 160501), the Princeton Institutional Review Board, and the NYU Institutional Review Board. Kirk Bansak, Rafaela Dancygier, Dominik Hangartner, Daniel Hopkins, and Yotam Margalit provided important feedback on the design.

†Associate Professor, Department of Political Science, UCSD. Email:cadida@ucsd.edu.

‡Post-doctoral research associate, Department of Politics, Princeton University. Email:adelinel@princeton.edu.

§Assistant Professor, Department of Political Science, NYU-Abu Dhabi. Email: mplatas@nyu.edu.

3. The instrument used is a survey experiment, including behavioral as well as attitudinal measures.

4. The survey will measure short-term and long-term effects of our treatments.

We are primarily interested in evaluating the extent to which individuals can be moved to adopt more inclusionary attitudes toward refugees as well as – secondarily – less discriminatory attitudes toward Muslims. Previous work on attitudes toward immigrants informs us that cultural threats have a significant effect on immigrant exclusion (Hainmueller and Hopkins, 2014, 2015; Sniderman, Hagendoorn and Prior, 2004). Our study hones in on one aspect of cultural threat of particular salience today: the refugee's religious identity as Muslim or Christian. Previous work also informs us that economic threats may play some role,[1] and we control for this in our design.

Our study follows Facchini, Margalit and Nakata (2016) in testing the extent to which immigration attitudes and behaviors can be moved. According to Facchini et al., "a key question that arises is whether public views on immigration predominantly reflect an instinctive, gut reaction toward the entry and presence of foreigners, or whether immigration attitudes are often formed by a more considered assessment of its benefits and costs." We ask this question in the context of the current Syrian refugee crisis, against which political elites in the U.S. have positioned themselves drawing largely on emotional appeals – such as the fear of terrorism. Our intervention experimentally manipulates respondents' exposure to an empathy prime vs. an informational treatment, and tests the effects on respondents' attitudes toward refugees, toward refugee policy, and toward Islamophobia; finally, we assess the persistence of effects over time.

2 Research Design

Relying on YouGov, we are procuring a nationally-representative sample of American adult citizens. Fielding began on Thursday, October 20, 2016. It is ongoing. No data was delivered to us as of the date of registration of this PAP. In this section, we outline our power calculation, randomization strategy, treatments, and full survey instrument.

[1] Although Hainmueller and Hopkins (2014) have called the economic threat hypothesis a "zombie hypothesis," other work finds significant effects of economic competition on anti-immigrant outcomes (Dancygier, 2010).

2.1 Randomization strategy

The design is shown in Figure 1 below. We note that the sample will be divided into two. A random half will complete the conjoint+DVs during Wave 1. The other random half will be invited to complete the conjoint+DVs during Wave 2, exactly seven days later.[2] Note that this is not a panel study. Rather, some respondents are randomly assigned to answer our outcome variable questions immediately; others are randomly assigned to respond to these questions seven days later (our aim is to complete data collection before the 2016 general election). We will remove all respondents who complete the survey on or after the 2016 U.S. general elections from main and secondary hypotheses as well as for robustness checks, and note when these respondents for the purpose of exploratory analysis.

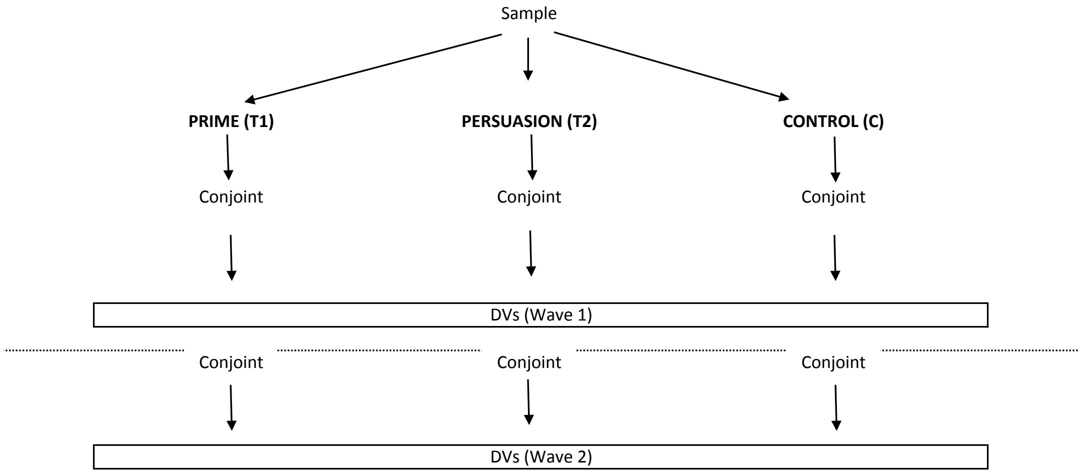

Figure 1: Randomization strategy

[2]Note that respondents may or may not complete the survey on the day they receive the invitation.

2.2 Treatments

Our instrument will be divided into four sections: the collection of pretreatment covariates, the administration of the treatment, the administration of the conjoints, and the collection of other outcome data. Our control condition will offer no treatment, such that it will consist of only three sections: the collection of pretreatment covariates, the administration of the conjoint, and the collection of other outcome data. The treatment conditions will be as follows:

1. Empathy prime: Albertson and Gadarian (2013, 2015*a*, 2015*b*) inform us that anxiety is a strong emotion driving political attitudes. When politicians instill fear and anxiety in us, they shape our political preferences. We ask whether the inverse is possible: what happens if we instill empathy rather than anxiety? Research has shown that this can be a fruitful approach for reducing prejudice toward transgender people (Broockman and Kalla 2016). Drawing from a real lesson plan designed by the Pulitzer Center (`http://pulitzercenter.org/builder/lesson/16023`), this prime will ask respondents the following open-ended questions before administering the conjoint: Imagine that you are a refugee fleeing persecution in a war-torn country.

    - What would you take with you, limited only to what you can carry yourself, on your journey?
    - Where would you flee to or would you stay in your home country?
    - What do you feel would be the biggest challenge for you?

    Note that this treatment must be taken as an encouragement treatment: we cannot actually measure whether or not we have instilled empathy.

2. Persuasion: In this experimental condition, we test whether better information about refugees has any effect on refugee inclusion. Indeed, Facchini et al. (2016) show that attitudes toward immigrants in Japan can be changed with a mass education campaign. In the case of the Syrian refugee crisis, one of the most salient arguments we have seen in the US context is how large of a contribution each country has made relative to its size. For example, the U.S. may have originally committed itself to admitting 10,000 Syrian refugees, but this represents only 0.003% of its population. By contrast, Canada originally pledged to host 25,000 Syrian refugees, or 0.07% of its population. Therefore, our persuasion treatment will present the following graph:

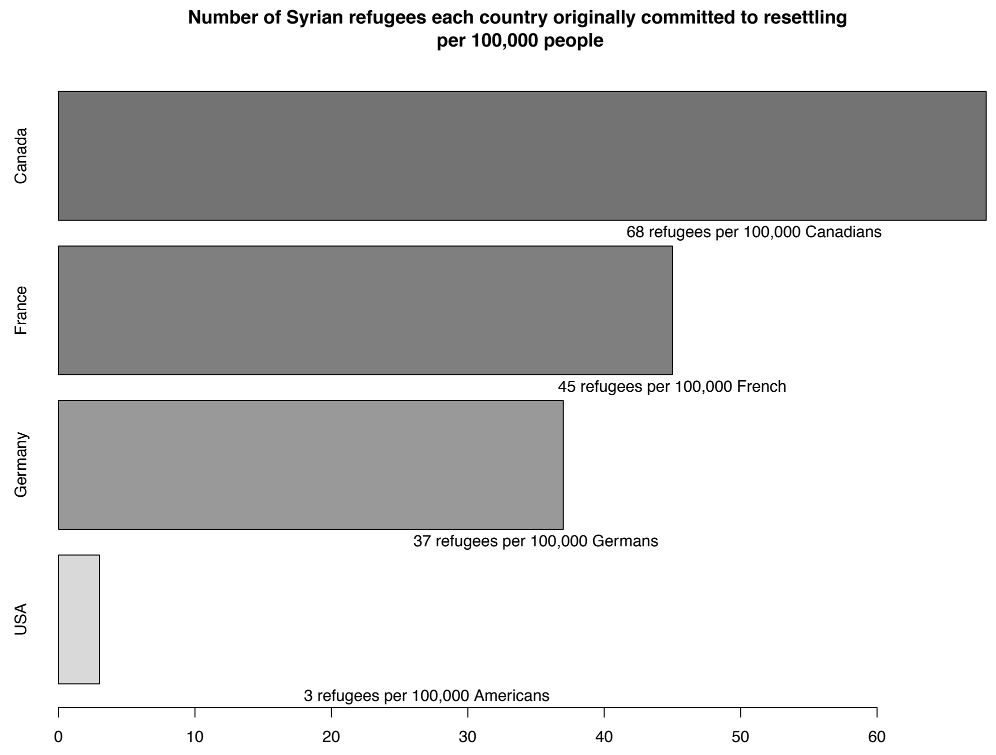

Then, respondents will be asked if this is new information to them or not, and asked whether they thought the U.S. had committed to relatively more, fewer, or the same number of refugees as the other countries. Finally, respondents will be asked to respond to the following prompt: "In a few sentences, please tell us how this information makes you feel about the US's current level of commitment to resettling refugees."

2.3 Survey Components

1. Pre-treatment covariates of respondent: gender, age, race, ethnicity, place of birth, education level, occupation, religion, religiosity, party ID, ethnocentrism, knowledge about US commitment to Syrian refugees. Note that gender, age, education level, religion, and party ID are standard questions in the YouGov panel and will thus not be repeated here. Note also that the ethnocentrism measure is adapted from the original Neuliep and McCroskey (1997) measure, adjusted to 8 items.[3]

2. Treatment:

[3]The original measure includes 24 items, each weighted equally. For eight items, each weighted equally with 5 points each, we will consider anyone scoring above 27 as high ethnocentric and anyone scoring below 17 as low ethnocentric, which is directly scaled from the original scoring scheme.

a. Control receives no pre-conjoint manipulation

b. Treatment 1 receives Empathy prime

c. Treatment 2 receives Persuasion prime

3. Conjoint

a. Each respondent receives three pairs of refugee profile comparisons (3 conjoints), sequentially.

b. Conjoint dimensions:

i. Country (Syria)

ii. Gender (M/F)

iii. Religion (Christian/Muslim)

iv. Previous occupation (farmer/teacher/doctor)

v. English fluency (fluent/broken/interpreter)

vi. Age (20/40/60)

4. Outcome measures

Y1 If you had to choose between them, which of these two refugees should be given priority to come to the United States to live?

Y2 On a scale from 1 to 7, where 1 indicates the United States should absolutely not admit the refugee and 7 indicates that the United States should definitely admit the refugee, how would you rate Refugee 1? (Wave 1)

Y3 Using the same scale, how would you rate Refugee 2?

Y4 Do you favor or oppose the U.S. taking in Syrian refugees who pass a government security screening?

Y5 Debriefing question: If you had to describe the goal of this survey to a friend, what would you describe it as?

Y6 Sending an anonymous letter to the next President of the U.S. in support of resettling refugees. (Behavioral question)

2.4 Main outcomes of interest

Following the studies on immigrant exclusion and Facchini et al.'s (2016) work on immigrant inclusion, we consider whether there is room to shift respondent opposition (or less favor)

towards Syrian refugees ($Y2$, $Y3$) as well as willingness to send an anonymous letter to the next President of the U.S. in support of resettling refugees ($Y6$).

Specifically, we explore whether there are effects of guiding respondents through an empathy exercise (T1) or a persuasive argument on the proportional burden of refugees the United States has committed to in comparison to similar Western countries (T2) on $Y2$, $Y3$ and $Y6$. For analysis purposes we can combine $Y2$ and $Y3$ as two different observations on the same outcome. To that effect, we refer to $Y2/Y3$ as simply $Y2$ continuing forward unless otherwise specified. We expect there to be positive effects of both treatment types on $Y2/Y3$ and $Y6$, and for these effects to persist (we hypothesize treatment effects to be captured in Wave 2). We also consider in our primary hypothesis section whether there is a heterogeneous treatment effect that is positive for both treatments with a Muslim refugee profile on the admittance of the refugee ($Y2$).

Below are our main outcomes of interest:

Y2 *On a scale from 1 to 7, where 1 indicates the United States should absolutely not admit the refugee and 7 indicates that the United States should definitely admit the refugee, how would you rate Refugee 1/2?*

$Y2$ and $Y3$ are numeric variables taking values from 1 to 7.

Y6 *Behavioral question: send an anonymous letter to the next President of the U.S. in support of resettling refugees*

$Y6$ is a binary variable where sending an anonymous letter is coded as 1 (This question asks the respondent if s/he is willing to send a comment in a letter we will compile and send to the next President. If the respondent answers "yes", and then fills out a sensical comment, s/he is coded as "1") and not sending is coded as 0.

2.5 Main predictors of interest

The main independent variables are Treatment 1 (Empathy treatment) and Treatment 2 (Persuasion treatment). We expect both types of treatments to have positive effects on our main outcomes of interest, $Y2$ and $Y6$ both in Wave 1 and in Wave 2.[4] We also hypothesize a possible positive heterogeneous treatment effect between the treatments and whether the refugee profile presented in the conjoint indicates that the refugee is Muslim on $Y2$. We code whether the refugee is Muslim or Christian as the variable 'M', where $M = 1$ if the refugee is Muslim and 0 otherwise. We can consider such a positive heterogeneous treatment effect with

[4]For $Y2$ and $Y3$ outcomes we will cluster standard errors by respondent.

a 'Muslim' refugee profile of particular substantive importance in light of the negative biases held against Muslims by Americans ((Panagopoulos, 2006), (Kalkan, Layman and Uslaner, 2009), (Savelkoul et al., 2011)), as documenting such an effect would provide supportive evidence that such negative biases can be ameliorated through empathy exercises and/or persuasive arguments.

2.6 Hypotheses

We present our hypotheses below. A full mapping of hypotheses, outcomes, and specifications can be found in Table 1.

Primary hypotheses

We test the differences in outcomes (Y2 and Y6) between each of the treatments and our control group. For the heterogeneous treatment effect of the Muslim profile and either T1 or T2, we specify a linear model but plan on conducting diagnostic tests suggested in Hainmueller et al. (2014) to determine whether our interaction specification satisfies linearity and common support assumptions. If not, we use Hainmueller et al.'s proposed kernel estimator.

For all hypotheses, we report a main specification without controls and one with the following controls, captured by the vector **X**: gender, age, US born, education level, religion, party ID, and ethnocentrism. We follow Lin (2013) and use the demeaning for **X** construction as well as interactions with the treatment to control for covariates (e.g. $Y = T + (X - \bar{X}) + T * (X - \bar{X})$). For the purposes of clarity below, however, we simply present the regressions in the conventional $Y = T$ formulation in the estimating equations to follow. We will also run the same specifications with de-meaned covariates and their interactions with the treatments but do not present the equations here. Again, for $Y2$ outcomes we cluster errors by respondent.

H1a (Empathy effect: score) Respondents who receive the empathy prime will give higher admission scores (7 point scale) than those in the control group. That is, $\beta_1 > 0$ in the below estimating equation:

$$Y2 = \alpha_0 + \beta_1 T1 + \epsilon \tag{1}$$

where α_0 is the intercept.

H1b (Persuasion effect: score) Respondents who receive the persuasion treatment will give higher admission scores than those in the control group. That is, $\beta_1 > 0$ in the below

estimating equation:

$$Y2 = \alpha_0 + \beta_1 T2 + \epsilon \tag{2}$$

where α_0 is the intercept.

H2a (Empathy effect: letter) Respondents receiving the empathy prime will be more likely than those in the control group to send an anonymous letter to the next President of the U.S. in support of resettling refugees. That is, $\beta_1 > 0$ in the below estimating equation:

$$Y6 = \alpha_0 + \beta_1 T1 + +\epsilon \tag{3}$$

where α_0 is the intercept.

H2b (Persuasion effect: letter) Respondents who receive the persuasion treatment will be more likely than those in the control group to send an anonymous letter to the next President of the U.S. in support of resettling refugees. That is, $\beta_1 > 0$ in the below estimating equation:

$$Y6 = \alpha_0 + \beta_1 T2 + \epsilon \tag{4}$$

where α_0 is the intercept.

H3a (Long term empathy effect: score) The empathy prime effect on improving scores given to refugee profiles may degrade over time, but will persist. That is, $\beta_1 > 0$ in the below estimating equation:

$$Y2' = \alpha_0 + \beta_1 T1 + \epsilon \tag{5}$$

where α_0 is the intercept, $Y2'$ is the measurement of $Y2$ at Wave 2.

H3b (Long term persuasion effect: score) The persuasion effect on improving scores given to refugee profiles may degrade over time, but will persist. That is, $\beta_1 > 0$ in the below estimating equation:

$$Y2' = \alpha_0 + \beta_1 T2 + \epsilon \tag{6}$$

where α_0 is the intercept, $Y2'$ is the measurement of $Y2$ at Wave 2.

H4a (Long term empathy effect: letter) The empathy prime effect on sending an anonymous letter to the next President of the U.S. in support of resettling refugees may degrade over time, but will persist. That is, $\beta_1 > 0$ in the below estimating equation:

$$Y6' = \alpha_0 + \beta_1 T1 + \epsilon \tag{7}$$

where α_0 is the intercept, $Y6'$ is the measurement of $Y6$ at Wave 2.

H4b (Long term persuasion effect: letter) The persuasion effect on sending an anonymous letter to the next President of the U.S. in support of resettling refugees may degrade over time, but will persist. That is, $\beta_1 > 0$ in the below estimating equation:

$$Y6' = \alpha_0 + \beta_1 T2 + \epsilon \tag{8}$$

where α_0 is the intercept, $Y6'$ is the measurement of $Y6$ at Wave 2.

Secondary hypotheses

As a set of secondary hypotheses, we examine heterogeneous treatment effects on $Y2$ for those who view a Muslim profile. For behavioral outcome $Y6$ we consider whether the number of Muslim profiles seen by respondents correlates with a larger Muslim "penalty". For this, we construct a variable C consisting of the number of Muslim profiles out of a total of 6 profiles (three sets of two profiles) presented to the respondent, where $C \in [0, 6]$.

H5a (Heterogeneous effect of empathy and Muslim: score) The empathy prime interacted with a Muslim refugee profile will lead to higher admission scores on average than no treatment interacted with Muslim refugee profiles. That is, $\beta_3 > 0$ in the below estimating equation:

$$Y2 = \alpha_0 + \beta_1 T1 + \beta_2 M + \beta_3 T1 * M + \epsilon \tag{9}$$

where α_0 is the intercept, and M is a binary variable for whether the refugee profile is Muslim.

H5b (Heterogeneous effect of persuasion and Muslim: score) The persuasion treatment will improve scores for Muslim refugee profiles and will be correlated with higher admission scores than Muslim refugee profiles in the control. That is, $\beta_3 > 0$ in the below estimating equation:

$$Y2 = \alpha_0 + \beta_1 T2 + \beta_2 M + \beta_3 T2 * M + \epsilon \tag{10}$$

where α_0 is the intercept, M is a binary variable for whether the refugee profile is Muslim.

H6a (Heterogeneous effect of empathy and Muslim: letter) The empathy prime combined with seeing Muslim refugee profiles will be correlated with a higher likelihood of sending an anonymous letter to the next President of the U.S. in support of resettling refugees,

controlling for the number of Muslim refugee profiles seen. That is, $\beta_3 > 0$ in the below estimating equation:

$$Y6 = \alpha_0 + \beta_1 T1 + \beta_2 C + \beta_3 T1 * C + \epsilon \tag{11}$$

where α_0 is the intercept, C is the number of Muslim refugee profiles presented.

H6b (Heterogeneous effect of persuasion and Muslim: letter) The persuasion prime combined with seeing Muslim refugee profiles will be correlated with a higher likelihood of sending an anonymous letter to the next President of the U.S. in support of resettling refugees, controlling for the number of Muslim refugee profiles seen. That is, $\beta_3 > 0$ in the below estimating equation:

$$Y6 = \alpha_0 + \beta_1 T2 + \beta_2 C + \beta_3 T2 * C + \epsilon \tag{12}$$

where α_0 is the intercept, C is the number of Muslim refugee profiles presented.

Additional hypotheses: robustness checks and verifications

Of secondary importance, we explore several further questions. First, we will verify whether there is evidence in support of what has already been well-established in the American politics literature — a Muslim "penalty" in inclusionary attitudes of Americans for an additional set of outcomes. We cluster errors by respondent for outcomes on $Y1$, $Y2$ and $Y3$.

H7a Given a choice between choosing a Christian refugee profile versus a Muslim refugee profile, respondents are on average more likely to prefer the Christian refugee profile. That is, $\beta_1 < 0$ in the below estimating equation:

$$Y1 = \alpha_0 + \beta_1 M + \epsilon \tag{13}$$

H7b Respondents will give lower admission scores on average to Muslim refugees relative to Christian refugees ("Muslim penalty"). That is, $\beta_1 < 0$ in the below estimating equation:

$$Y2 = \alpha_0 + \beta_1 M + \epsilon \tag{14}$$

H7c The likelihood of respondents sending an anonymous letter to the White House is negatively correlated with the number of Muslim refugee profiles presented to them.

That is, $\beta_1 < 0$ in the below estimating equation:

$$Y6 = \alpha_0 + \beta_1 C + \epsilon \tag{15}$$

Next, we consider long-term heterogeneous treatment effects of viewing Muslim profiles.

H8a The heterogeneous effect of the empathy treatment with the ratings of a Muslim refugee profile compared to a Christian refugee profile may degrade in magnitude over time but will persist. $\beta_3 > 0$ in the below estimating equation:

$$Y_2' = \alpha_0 + \beta_1 T_1 + \beta_2 M + \beta_3 T_1 * M + \epsilon \tag{16}$$

where Y_2' is the measurement of $Y2$ at Wave 2.

H8b The heterogeneous effect of the persuasion treatment with the ratings of a Muslim refugee profile compared to a Christian refugee profile may degrade in magnitude over time but will persist. $\beta_3 > 0$ in the below estimating equation:

$$Y_2' = \alpha_0 + \beta_1 T_2 + \beta_2 M + \beta_3 T_2 * M + \epsilon \tag{17}$$

where Y_2' is the measurement of $Y2$ at Wave 2.

H9a The heterogeneous effect of the empathy treatment with seeing Muslim refugee profiles on sending an anonymous letter to the White House may degrade in magnitude over time but will persist. $\beta_3 > 0$ in the below estimating equation:

$$Y_6' = \alpha_0 + \beta_1 T_1 + \beta_2 C + \beta_3 T_1 * C + \epsilon \tag{18}$$

where Y_6' is the measurement of $Y6$ at Wave 2.

H9b The heterogeneous effect of the persuasion treatment with seeing Muslim refugee profiles on sending an anonymous letter to the White House may degrade in magnitude over time but will persist. $\beta_3 > 0$ in the below estimating equation:

$$Y_6' = \alpha_0 + \beta_1 T_2 + \beta_2 C + \beta_3 T_2 * C + \epsilon \tag{19}$$

where Y_6' is the measurement of $Y6$ at Wave 2.

As exploratory analysis, we consider whether there might be a heterogeneous treatment effect of our empathy/persuasion treatments with whether a refugee profile is Muslim on

$Y4$, the inclusionary attitude for the U.S. taking in Syrian refugees who pass a government security screening. We use our constructed C variable for these exploratory tests.

H10a (Heterogeneous effect of empathy and Muslim: screening) The empathy prime combined with seeing Muslim refugee profiles will be correlated with a higher likelihood of favoring the U.S. taking in Syrian refugees who pass a government security screening relative to the control, controlling for the number of Muslim refugee profiles seen. That is, $\beta_3 > 0$ in the below estimating equation:

$$Y4 = \alpha_0 + \beta_1 T1 + \beta_2 C + \beta_3 T1 * C + \epsilon \tag{20}$$

where α_0 is the intercept, C is the number of Muslim refugee profiles presented.

H10b (Heterogeneous effect of persuasion and Muslim: screening) The persuasion prime combined with seeing Muslim refugees profile will be correlated with a higher likelihood of favoring the U.S. taking in Syrian refugees who pass a government security screening than in the control, controlling for the number of Muslim refugee profiles seen. That is, $\beta_3 > 0$ in the below estimating equation:

$$Y4 = \alpha_0 + \beta_1 T1 + \beta_2 C + \beta_3 T2 * C + \epsilon \tag{21}$$

where α_0 is the intercept, C is the number of Muslim refugee profiles presented.

H11a The heterogeneous effect of the empathy treatment and seeing Muslim refugee profiles (compared to a Christian refugee profile) on favoring the U.S. taking in Syrian refugees who pass a government security screening may degrade in magnitude over time but will persist. $\beta_3 > 0$ in the below estimating equation:

$$Y_4' = \alpha_0 + \beta_1 T_1 + \beta_2 C + \beta_3 T_1 * C + \epsilon \tag{22}$$

where Y_4' is the measurement of $Y4$ at Wave 2.

H11b The heterogeneous effect of the persuasion treatment with and seeing a Muslim refugee profile compared to a Christian refugee profile on the likelihood of favoring the U.S. taking in Syrian refugees who pass a government security screening may degrade in magnitude over time but will persist. $\beta_3 > 0$ in the below estimating equation:

$$Y_4' = \alpha_0 + \beta_1 T_2 + \beta_2 C + \beta_3 T_2 * C + \epsilon \tag{23}$$

where Y_4' is the measurement of $Y4$ at Wave 2.

As a robustness check for our primary outcome $Y2$, we will check to see whether the empathy and persuasion treatments have similar positive effects on outcome $Y4$ that should reasonably have some correlations with $Y2$. We will also check for whether there is an overall reduction in discrimination on any basis in refugee pair profiles (reported differences in scores assigned to $Y2$ and $Y3$).

As we simply pooled responses to $Y2$ and $Y3$ amongst all respondents in the primary hypotheses section, we will also run a robustness check on the relevant estimating equations with respondent-level grouped errors (not detailed below).

H12a The empathy prime will significantly increase the respondent's reported inclusionary attitude for the U.S. taking in Syrian refugees who pass a government security screening. That is, $\beta_1 > 0$ in the below estimating equation:

$$Y4 = \alpha_0 + \beta_1 T1 + \epsilon \tag{24}$$

H12b The persuasion treatment will significantly increase the respondent's reported inclusionary attitude for the U.S. taking in Syrian refugees who pass a government security screening. That is, $\beta_1 > 0$ in the below estimating equation:

$$Y4 = \alpha_0 + \beta_1 T2 + \epsilon \tag{25}$$

H13a The empathy prime will significantly decrease the magnitude of the average difference in admission scores across refugee pairs (i.e. reduce discrimination on any basis).

$$Y_{2,3} = \alpha_0 + \beta_1 T1 + \epsilon \tag{26}$$

where $Y_{2,3}$ is the absolute difference between $Y2$ and $Y3$ in a given pair of refugee profiles: $|Y2 - Y3|$.

H13b The persuasion treatment will significantly decrease the magnitude of the average difference in admission scores across refugee pairs (i.e. reduce discrimination on any basis).

$$Y_{2,3} = \alpha_0 + \beta_1 T2 + \epsilon \tag{27}$$

where $Y_{2,3}$ is the absolute difference between $Y2$ and $Y3$ in a given pair of refugee profiles: $|Y2 - Y3|$.

Finally, although the study is not powered or designed to specifically detect differences between the empathy and persuasion treatment effects, as the literature does not have a

clear comparison between the two types of treatments as of this writing, we consider this exercise an exploratory analysis to shed some light on such a comparison.

H14a Respondents who receive the empathy prime will have significantly different point scores reported for refugee profiles than those in the persuasion group. That is, $\beta_1 \neq 0$ in the below estimating equation:

$$Y2 = \alpha_0 + \beta_1 T_{1,2} + \epsilon \tag{28}$$

where $T_{1,2}$ is a binary variable that takes the value 1 when the respondent is in the empathy group and 0 when the respondent is in the persuasion group.

H14b Respondents who receive the empathy prime will have a significantly different preference for the U.S. taking in Syrian refugees who pass a government security screening than respondents in the persuasion group. That is, $\beta_1 \neq 0$ in the below estimating equation:

$$Y4 = \alpha_0 + \beta_1 T_{1,2} + \epsilon \tag{29}$$

where $T_{1,2}$ is a binary variable that takes the value 1 when the respondent is in the empathy group and 0 when the respondent is in the persuasion group.

H14c The empathy prime group will have a significantly different preference compared to the persuasion group toward sending an anonymous letter to the White House. $\beta_1 \neq 0$ in the below estimating equation:

$$Y6 = \alpha_0 + \beta_1 T_{1,2} + \epsilon \tag{30}$$

where $T_{1,2}$ is a binary variable that takes the value 1 when the respondent is in the empathy group and 0 when the respondent is in the persuasion group.

H15a The effects of the empathy treatment on assigning higher point scores to refugee profiles will degrade significantly differently than the persuasion treatment over time. $\beta_1 \neq \beta_2$ in the below estimating equation:

$$Y_2' = \alpha_0 + \beta_1 T1 + \beta_2 T2 + \epsilon \tag{31}$$

where Y_2' is measurement of $Y2$ in Wave 2.

H15b The effects of the empathy treatment on favoring or opposing the U.S. taking in Syrian refugees will degrade significantly differently than the persuasion treatment over time.

$\beta_1 \neq \beta_2$ in the below estimating equation:

$$Y_4' = \alpha_0 + \beta_1 T1 + \beta_2 T2 + \epsilon \tag{32}$$

where Y_4' is measurement of $Y4$ in Wave 2.

H15c The effects of the empathy treatment on sending an anonymous letter to the White House will degrade significantly differently than the persuasion treatment over time. $\beta_1 \neq \beta_2$ in the below estimating equation:

$$Y_6' = \alpha_0 + \beta_1 T1 + \beta_2 T2 + \epsilon \tag{33}$$

where Y_6' is measurement of $Y6$ in Wave 2.

If respondents provide enough variation in text for their answers to $Y6$, we will conduct text analysis for major themes present in the texts. In particular, we will explore patterns and types of themes in what respondents would take with them if they were refugees and how they feel about the US commitment to Syrian refugees. We will also use the quantity of (non-nonsensical) text as another measure of compliance.

Finally, we will conduct a manipulation check by analyzing responses for $Y5$ to see if there is evidence of social desirability bias.

Table 1: Specifications, Hypotheses and Measures

Family	#	Abbreviated Hypothesis	Y	X	Interact'n	Subset	Spec'n
Primary	H1a	Empathy score	Y2	T1		T1a, C	Eq. 1
	H1b	Persuasion score	Y2	T2		T2a, C	Eq. 2
	H2a	Empathy letter	Y6	T1		T1a, C	Eq. 3
	H2b	Persuasion letter	Y6	T2		T2a, C	Eq. 4
	H3a	Longterm empathy score	Y2	T1		T1b, C	Eq. 5
	H3b	Longterm persuasion score	Y2	T2		T2b, C	Eq. 6
	H4a	Longterm empathy letter	Y6	T1		T1b, C	Eq. 7
	H4b	Longterm persuasion letter	Y6	T2		T2b, C	Eq. 8
Secondary	H5a	Empathy Muslim score	Y2*	T1	T1*M	T1a, C	Eq. 9
	H5b	Persuasion Muslim score	Y2*	T2	T1*M	T2a, C	Eq. 10
	H6a	Empathy Muslim number letter	Y6	T1	T1*C	T1a, C	Eq. 11
	H6b	Persuasion Muslim number letter	Y6	T2	T2*C	T2a, C	Eq. 12
Muslim penalty	H7a	Muslim penalty choice	Y1	M		All	Eq. 13
	H7b	Muslim penalty score	Y2	M		All	Eq. 14
	H7c	Muslim penalty letter	Y6	C		All	Eq. 15
	H8a	Empathy Muslim score persist	Y2*	T1	T1*M	T1b, C	Eq. 16
	H8b	Persuasion Muslim score persist	Y2*	T2	T1*M	T2b, C	Eq. 17
	H9a	Empathy Muslim number letter persist	Y6	T1	T1*C	T1b, C	Eq. 18
	H9b	Persuasion Muslim number letter persist	Y6	T2	T2*C	T2b, C	Eq. 19
	H10a	Empathy Muslim number screening	Y4	T1	T1*C	T1a, C	Eq. 20
	H10b	Persuasion Muslim number screening	Y4	T2	T2*C	T2a, C	Eq. 21
	H11a	Empathy Muslim number screening persist	Y4	T1	T1*C	T1b, C	Eq. 22
	H11b	Persuasion Muslim number screening persist	Y4	T2	T2*C	T2b, C	Eq. 23
Robustness	H12a	Empathy screening	Y4	T1		T1a, T1b, C	Eq. 24
	H12b	Persuasion screening	Y4	T2		T2a, T2b, C	Eq. 25
	H13a	Empathy score difference	$Y_{2,3}$	T1		T1a, T1b, C	Eq. 26
	H13b	Persuasion score difference	$Y_{2,3}$	T2		T2a, T2b, C	Eq. 27
Comparing treatments	H14a	Treatment difference scores	Y2	$T1, T2$		All	Eq. 28
	H14b	Treatment difference screening	Y4	$T1, T2$		All	Eq. 29
	H14c	Treatment difference letter	Y6	$T1, T2$		All	Eq. 30
	H15a	Treatment persistence scores	Y2	$T1, T2$		All	Eq. 31
	H15b	Treatment persistence screening	Y4	$T1, T2$		All	Eq. 32
	H15c	Treatment persistence letter	Y6	$T1, T2$		All	Eq. 33

Table 2: **Mapping of hypotheses, variables, and subsets of data**: the table presents the groups of hypotheses and estimating equations featured in this PAP. Associated variables and subsets of data are presented. For subsets of data, T1a (/T2a) refers to the subset of individuals who received Treatment 1 Empathy treatment (/Treatment 2 Persuasion treatment) and answered their dependent variable questions in Wave 1. T1b (/T2b) refers to individuals who received Treatment 1 (/Treatment 2) and answered their dependent variable questions in Wave 2. A * next to the outcome variable indicates we will estimate the equation on only the first set of conjoints a respondent views.

2.7 Power calculation

In the Facchini, Margalit and Nakata (2016) study, the authors found that the information treatment had between a 12 and 21 percentage point increase in approving acceptance of an immigrant; over a longer period of time, this effect tended to halve.

Our positive persuasion treatment is most similar to the Facchini et al. intervention and so we reflect our estimated treatment effect of the positive persuasion treatment accordingly. Our power calculations are designed to capture our primary hypotheses.

We consider a scenario where the short term positive persuasion treatment size is a 9.5% increase in the outcome variable, the short term empathy treatment size is 19.5%, and long term treatment effects of both treatments are merely half of the short term effect sizes. We also test two heterogeneous treatment effects, the persuasion treatment and a Muslim refugee profile interacted together, as well as an empathy treatment and Muslim refugee profile interacted together. We assume the Muslim effect to be -10, which is consistent with the difference between the average American's reported feelings for Muslims (in a thermometer measure) compared to the average American's reported feelings for an average American, as reported in Kalkan et al. (2009). We set the SD on the outcome to 35 in order to be conservative in our power calculations and require a power of 80% (this is also in the realm of the SD of the effect size uncovered by Hainmueller and Hopkins (2015)). For these tests, we would need a total sample size of $N = 5,300$ to detect treatment effects (compared to the control arm) for each treatment in both the short and long terms. See the appendix for the full R code used to generate these power calculations. Figure 2 below illustrates the changes in our sample size needs as we shift some of our assumptions:

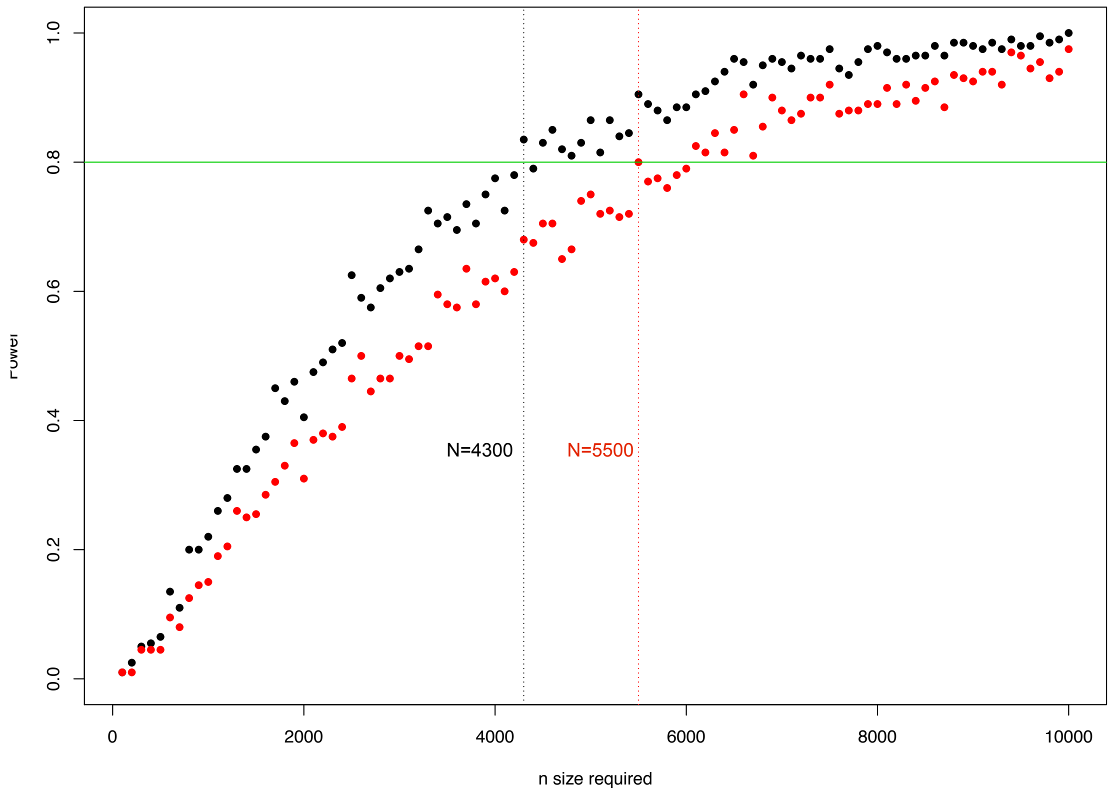

Figure 2: **Power calculations**. The x-axis depicts the sample size (n), while the y-axis represents the power achieved from 0 to 1. The green horizontal line is power at 0.8. Black scatter points are power calculations made with SD of the outcome variable set at 35, while red ones are power calculations made with SD set at 30; all other parameters were set to be the same (assumed treatment effects and heterogeneous treatment effects). The required n-size for power calculations made with SD set at 35 is 4,300, while the required n-size for power calculations made with SD=30 is 5,500.

3 Multiple comparisons

In order to address the problem of multiple comparisons, we restrict the number of primary hypotheses. We restrict ourselves to two major dependent variables ($Y1$, $Y6$), two treatments ($T1$, $T2$) for short and long terms, for a total of eight hypotheses. We also look at a set of secondary hypotheses for heterogeneous tests ($T1 * X$, $T2 * X$). For this, we have a total of four hypotheses. The outcome variables are not likely to be independent, so we take into account this dependency and use a rule of thumb $\alpha = 1 - (0.05)^{\frac{1}{\sqrt{h}}}$, a correction popularized by John Tukey (see Braun (1994)).

4 Survey Instrument

S1 Which of these statements best describes you?

- I am an immigrant to the USA and a naturalized citizen [continue to S3]
- I am an immigrant to the USA but not a citizen [respondent not eligible for this survey]
- I was born in the USA but at least one of my parents is an immigrant [continue to S2]
- My parents and I were born in the USA but at least one of my grandparents was an immigrant [continue to S2]
- My parents, grandparents and I were all born in the USA [continue to S2]

S2 In which state were you born? [scroll down all States/territories]

S3 In which country were you born? [scroll down all countries]

S4 How many Syrian refugees do you think have already been admitted into the United States since September 11, 2001? [Fewer than 1,000/About 5,000/About 10,000/More than 10,000]

S5 Over the past two years, do you think the U.S. has admitted proportionally more or fewer Syrian refugees than Canada? [More/Fewer]

S6 Over the past two years, do you think the U.S. has admitted proportionally more or fewer Syrian refugees than France? [More/Fewer]

S7 Over the past two years, do you think the U.S. has admitted proportionally more or fewer Syrian refugees than Germany? [More/Fewer]

S8 Do you agree or disagree with the following statements? [Str. disagree/Disagree/Neutral/Agree/Str. agree]

- Most other cultures are backward compared with my culture
- My culture should be the role model for other cultures
- People from other cultures act strangely when they come to my culture
- Lifestyles in other cultures are just as valid as are those in my culture
- Other cultures should try to be more like my culture

- I am not interested in the values and customs of other cultures
- People in my culture could learn a lot from people from other cultures
- Most people from other cultures just don't know what's good for them

---

S9 Randomization into equal thirds: Control [SC], Empathy [SE], Persuasion [SP]

SC0 Randomization of SC sample into equal halves. One half gets the below questions right away. The other half gets the below questions 1 week later.

SC1 Imagine that you are an official making decisions about which refugees to let into the United States. On the next few pages, you will see descriptions of two refugees from Syria and then you will be asked a set of questions about them. Please read the descriptions of the refugees carefully.

- Country (Syria)
- Gender (Male/Female)
- Religion (Christian/Muslim)
- Job before leaving Syria (Farmer/Teacher/Doctor)
- English fluency (This applicant speaks fluent English/ This applicant speaks broken English/This applicant speaks no English and needed an interpreter)
- Age (20/40/60)

SC2 Imagine that you are an official making decisions about which refugees to let into the United States. Please read the descriptions of the potential refugees carefully. These refugees all come from Syria. Please indicate which of the two Syrian refugees you would personally prefer to see admitted to the United Sttates [Refugee 1/Refugee 2]

SC3 On a scale from 1 to 7, where 1 indicates the United States should absolutely not admit the refugee and 7 indicates that the United States should definitely admit the refugee, how would you rate Refugee 1? [1-7]

SC4 Using the same scale, how would you rate Refugee 2? [1-7] [REPEAT SC1 THROUGH SC4 TWICE FOR A TOTAL OF THREE CONJOINTS]

SC5 Do you favor or oppose the U.S. taking in Syrian refugees who pass a government security screening? [Favor/Oppose]

SC6 If you had to describe the goal of this survey to a friend, what would you describe it as? [open-ended]

SC7 The next President of the United States will have important decisions to make about how to deal with the Syrian refugee crisis. Would you be interested in expressing

your support for refugees to the next president of the United States in an anonymous forum? [No, thank you/Yes, I am interested]

SC8 (If yes above) Please use the space below to express your support of refugees to the next president of the United States. The research team will compile these entries and submit them in a letter to the president after he or she is inaugurated in 2017. Your response will be completely anonymous. [Open ended/Decline]

SE0 Randomization of SE sample into equal halves. One half gets all the below questions right away. The other half gets SE1 right away, and the rest 1 week later.

SE1 Imagine that you are a refugee fleeing persecution in a war-torn country. In the pages that follow, you will be asked a set of questions about how you imagine this experience would be like for you. You will have space to provide written responses. Please answer each question with as much detail as you can.

- Imagine that you are a refugee fleeing persecution in a war-torn country. What would you take with you, limited only to what you can carry yourself, on your journey? [open-ended]
- Imagine that you are a refugee fleeing persecution in a war-torn country. Where would you flee to or would you stay in your home country? [open-ended]
- Imagine that you are a refugee fleeing persecution in a war-torn country. What do you feel would be the biggest challenge for you? [open-ended]

SE2 Imagine that you are an official making decisions about which refugees to let into the United States. On the next few pages, you will see descriptions of two refugees from Syria and then you will be asked a set of questions about them. Please read the descriptions of the refugees carefully.

- Country (Syria)
- Gender (Male/Female)
- Religion (Christian/Muslim)
- Job before leaving Syria (Farmer/Teacher/Doctor)
- English fluency (This applicant speaks fluent English/ This applicant speaks broken English/This applicant speaks no English and needed an interpreter)
- Age (20/40/60)

SE3 Imagine that you are an official making decisions about which refugees to let into the United States. Please read the descriptions of the potential refugees carefully. These refugees all come from Syria. Please indicate which of the two Syrian refugees you would personally prefer to see admitted to the United States [Refugee 1/Refugee 2]

SE4 On a scale from 1 to 7, where 1 indicates the United States should absolutely not admit the refugee and 7 indicates that the United States should definitely admit the refugee, how would you rate Refugee 1? [1-7]

SE5 Using the same scale, how would you rate Refugee 2? [1-7] [REPEAT SE2 THROUGH SE5 TWICE FOR A TOTAL OF THREE CONJOINTS]

SE6 Do you favor or oppose the U.S. taking in Syrian refugees who pass a government security screening? [Favor/Oppose]

SE7 If you had to describe the goal of this survey to a friend, what would you describe it as? [open-ended]

SE8 The next President of the United States will have important decisions to make about how to deal with the Syrian refugee crisis. Would you be interested in expressing your support for refugees to the next president of the United States in an anonymous forum? [No, thank you/Yes, I am interested]

SE9 (If yes above) Please use the space below to express your support of refugees to the next president of the United States. The research team will compile these entries and submit them in a letter to the president after he or she is inaugurated in 2017. Your response will be completely anonymous. [Open ended/Decline]

SP0 Randomization of SP sample into equal halves. One half gets all the below questions right away. The other half gets SP1 right away, and the rest 1 week later.

SP1 Did you know? The United States has actually committed to resettling a much smaller number of refugees than has France, Germany, or Canada. The figure below shows how many refugees each of the following countries originally pledged to resettle per 100,000 people. [Show below figure]

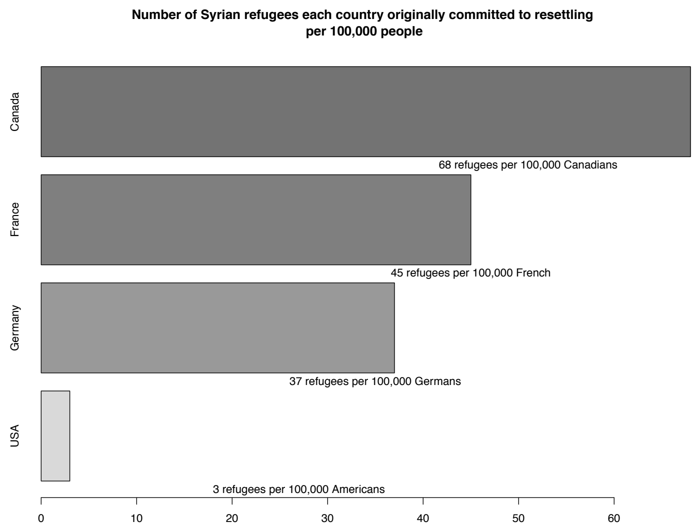

(a) Is this new information for you?

(b) Prior to viewing this figure, did you believe the US had committed to resettling proportionally more, fewer, or about the same amount of refugees as other democracies such as Canada, France, or Germany? [More/Fewer/Same]

(c) In a few sentences, please tell us how this information makes you feel about the US's level of commitment to resettling refugees [Open-ended]

SP2 Imagine that you are an official making decisions about which refugees to let into the United States. On the next few pages, you will see descriptions of two refugees from Syria and then you will be asked a set of questions about them. Please read the descriptions of the refugees carefully.

- Country (Syria)
- Gender (Male/Female)
- Religion (Christian/Muslim)
- Job before leaving Syria (Farmer/Teacher/Doctor)
- English fluency (This applicant speaks fluent English/ This applicant speaks broken English/This applicant speaks no English and needed an interpreter)
- Age (20/40/60)

SP3 Imagine that you are an official making decisions about which refugees to let into the United States. Please read the descriptions of the potential refugees carefully. These refugees all come from Syria. Please indicate which of the two Syrian refugees you would personally prefer to see admitted to the United States [Refugee 1/Refugee 2]

SP4 On a scale from 1 to 7, where 1 indicates the United States should absolutely not admit the refugee and 7 indicates that the United States should definitely admit the refugee, how would you rate Refugee 1? [1-7]

SP5 Using the same scale, how would you rate Refugee 2? [1-7] [REPEAT SP2 THROUGH SP5 TWICE FOR A TOTAL OF THREE CONJOINTS]

SP6 Do you favor or oppose the U.S. taking in Syrian refugees who pass a government security screening? [Favor/Oppose]

SP7 If you had to describe the goal of this survey to a friend, what would you describe it as? [open-ended]

SP8 The next President of the United States will have important decisions to make about how to deal with the Syrian refugee crisis. Would you be interested in expressing your support for refugees to the next president of the United States in an anonymous forum? [No, thank you/Yes, I am interested]

SP9 (If yes above) Please use the space below to express your support of refugees to the next president of the United States. The research team will compile these entries and submit them in a letter to the president after he or she is inaugurated in 2017. Your response will be completely anonymous. [Open ended/Decline]

5 Appendix

5.1 Power calculation R code

```
### Power calculations ###
# Author: Adeline Lo
# Maintained: AL
# Date: Aug 31, 2016
##########################

# C, T1=(+) persuasion short, T2=empathy short, T3=(+) persuasion
    long, T4=empathy long
# M=Muslim covariate (binary 0/1) which will interact with T1 and
    T2
rm(list=ls())
#install.packages("randomizr")
library(randomizr)    # randomizr package for complete random
    assignment

possible.ns <- seq(from=100, to=10000, by=100)
power.shorttreatments <- rep(NA, length(possible.ns)) #T1 and T2
power.persuasion<- rep(NA, length(possible.ns)) #T1 and T3
power.empathy<- rep(NA, length(possible.ns)) #T2 and T4
power.persuasionM <- rep(NA, length(possible.ns)) #T1*M
power.empathyM <- rep(NA, length(possible.ns)) #T2*M

power.alltreatments <- rep(NA,length(possible.ns)) #T1, T2, T3, T4
    , T1*M, T2*M
power.fullranking <- rep(NA, length(possible.ns))
alpha <- 0.1 #(one-tailed test at .05 level)
sims <- 200

## Where we hold p-values and means
  p.T1vsC <- rep(NA, sims) #pvalues
  p.T2vsC <- rep(NA, sims)
  p.T3vsC <- rep(NA, sims)
```

```
  p.T4vsC <- rep(NA, sims)
  p.T1MvsC <- rep(NA, sims) #interactions
  p.T2MvsC <- rep(NA, sims)
  p.T2vsT1 <- rep(NA, sims) #difference between T1/T2 in short run
  p.T3vsT1 <- rep(NA, sims) #persistence of T1
  p.T4vsT2 <- rep(NA, sims) #persistence of T2
  p.T4vsT3 <- rep(NA, sims) #difference between T1/T2 in long run

    c.T1vsC <- rep(NA, sims) #means
    c.T2vsC <- rep(NA, sims)
    c.T3vsC <- rep(NA, sims)
    c.T4vsC <- rep(NA, sims)
    c.T1MvsC <- rep(NA, sims) #interactions
    c.T2MvsC <- rep(NA, sims) #interactions
    c.T2vsT1 <- rep(NA, sims) #difference between T1/T2 in short
       run
    c.T3vsT1 <- rep(NA, sims) #persistence of T1
    c.T4vsT2 <- rep(NA, sims) #persistence of T2
    c.T4vsT3 <- rep(NA, sims) #difference between T1/T2 in long
       run
# Treatment effects assumed
tau_1 = 9.5   #treatment effect 1 persuasion (+) short
tau_2 = 19.5   #treatment effect 3 empathy
tau_3 = tau_1*0.5 #longterm effect of T1
tau_4 = tau_2*0.5 #longterm effect of T2

set.seed(831)#aug 31

#### Outer loop to vary the number of subjects ####
for (j in 1:length(possible.ns)){
  N <- possible.ns[j]
  #### Inner loop to conduct experiments "sims" times over for
     each N ####
  for (i in 1:sims){
```

```
M <- c(rep("M", N/2), rep("C", N/2))        # Generate "Muslim/Christian" covariate
effectM <- -10                              # Hypothesize the "effect" of muslim on outcome, from Kalkan et al. 2009, Muslim feelings therm 52.2, overall pop 62.8

## Hypothesize Control Outcome
Y0 <-  rnorm(n=N, mean=50, sd=30)  #
Y1 = Y0 + tau_1
Y2 = Y0 + tau_2
Y3 = Y0 + tau_3
Y4 = Y0 + tau_4
Y5 = Y0 + effectM*(M=="M") + tau_1*(effectM*(M=="M")) # interaction term with T1
Y6 = Y0 + effectM*(M=="M") + tau_2*(effectM*(M=="M")) # interaction term with T2
Z.sim <- complete_ra(N=N, num_arms=7)
Y.sim <- Y0*(Z.sim=="T7") + Y1*(Z.sim=="T1") + Y2*(Z.sim=="T2") + Y3*(Z.sim=="T3") + Y4*(Z.sim=="T4")
          + Y5*(Z.sim=="T5")+Y6*(Z.sim=="T6")
frame.sim <- data.frame(Y.sim, Z.sim)
fit.T1vsC.sim <- lm(Y.sim ~ Z.sim=="T1", data=subset(frame.sim, (Z.sim!="T2"&Z.sim!="T3"&Z.sim!="T4"&Z.sim!="T5"&Z.sim!="T6")))
fit.T2vsC.sim <- lm(Y.sim ~ Z.sim=="T2", data=subset(frame.sim, (Z.sim!="T1"&Z.sim!="T3"&Z.sim!="T4"&Z.sim!="T5"&Z.sim!="T6")))
fit.T3vsC.sim <- lm(Y.sim ~ Z.sim=="T3", data=subset(frame.sim, (Z.sim!="T1"&Z.sim!="T2"&Z.sim!="T4"&Z.sim!="T5"&Z.sim!="T6")))
fit.T4vsC.sim <- lm(Y.sim ~ Z.sim=="T4", data=subset(frame.sim, (Z.sim!="T1"&Z.sim!="T2"&Z.sim!="T3"&Z.sim!="T5"&Z.sim!="T6")))
fit.T1MvsC.sim <- lm(Y.sim ~ Z.sim=="T5", data=subset(frame.sim, (Z.sim!="T1"&Z.sim!="T2"&Z.sim!="T3"&Z.sim!="T4"&Z.sim!="T6"))) #interactions TI*M
```

```
fit.T2MvsC.sim <- lm(Y.sim ~ Z.sim=="T6", data=subset(frame.
    sim, (Z.sim!="T1"&Z.sim!="T2"&Z.sim!="T3"&Z.sim!="T4"&Z.sim
    !="T5"))) #interactions TI*M

fit.T2vsT1.sim <- lm(Y.sim ~ Z.sim=="T2", data=subset(frame.
    sim, (Z.sim!="T3"&Z.sim!="T4"&Z.sim!="T5"&Z.sim!="T6"&Z.sim
    !="T7"))) #leave out 1,2
fit.T3vsT1.sim <- lm(Y.sim ~ Z.sim=="T3", data=subset(frame.
    sim, (Z.sim!="T2"&Z.sim!="T4"&Z.sim!="T5"&Z.sim!="T6"&Z.sim
    !="T7"))) #leave out 1,3
fit.T4vsT2.sim <- lm(Y.sim ~ Z.sim=="T4", data=subset(frame.
    sim, (Z.sim!="T1"&Z.sim!="T3"&Z.sim!="T5"&Z.sim!="T6"&Z.sim
    !="T7"))) #leave out 2,4
fit.T4vsT3.sim <- lm(Y.sim ~ Z.sim=="T4", data=subset(frame.
    sim, (Z.sim!="T1"&Z.sim!="T2"&Z.sim!="T5"&Z.sim!="T6"&Z.sim
    !="T7"))) #leave out 3,4
### Need to capture coefficients and pvalues (one-tailed tests
    , so signs are important)
c.T1vsC[i] <- summary(fit.T1vsC.sim)$coefficients[2,1]
c.T2vsC[i] <- summary(fit.T2vsC.sim)$coefficients[2,1]
c.T3vsC[i] <- summary(fit.T3vsC.sim)$coefficients[2,1]
c.T4vsC[i] <- summary(fit.T4vsC.sim)$coefficients[2,1]
c.T1MvsC[i] <- summary(fit.T4vsC.sim)$coefficients[2,1]
c.T2MvsC[i] <- summary(fit.T4vsC.sim)$coefficients[2,1]
        c.T2vsT1[i] <- summary(fit.T2vsT1.sim)$coefficients[2,1]
        c.T3vsT1[i] <- summary(fit.T3vsT1.sim)$coefficients[2,1]
        c.T4vsT2[i] <- summary(fit.T4vsT2.sim)$coefficients[2,1]
        c.T4vsT3[i] <- summary(fit.T4vsT3.sim)$coefficients[2,1]

p.T1vsC[i] <- summary(fit.T1vsC.sim)$coefficients[2,4]
p.T2vsC[i] <- summary(fit.T2vsC.sim)$coefficients[2,4]
p.T3vsC[i] <- summary(fit.T3vsC.sim)$coefficients[2,4]
p.T4vsC[i] <- summary(fit.T4vsC.sim)$coefficients[2,4]
p.T1MvsC[i] <- summary(fit.T4vsC.sim)$coefficients[2,4]
p.T2MvsC[i] <- summary(fit.T4vsC.sim)$coefficients[2,4]
        p.T2vsT1[i] <- summary(fit.T2vsT1.sim)$coefficients[2,4]
```

```
        p.T3vsT1[i] <- summary(fit.T3vsT1.sim)$coefficients[2,4]
        p.T4vsT2[i] <- summary(fit.T4vsT2.sim)$coefficients[2,4]
        p.T4vsT3[i] <- summary(fit.T4vsT3.sim)$coefficients[2,4]
}
#power.atleastone[j] <- mean(c.T1vsC>0 & c.T2vsC>0 & c.T3vsC>0 &
    c.T4vsC>0 & (p.T1vsC < alpha/2 | p.T2vsC < alpha/2 | p.T3vsC
    < alpha/2 | p.T4vsC < alpha/2))
#power.shortempathy[j] <-mean(c.T2vsC>0 & p.T2vsC < alpha/2)
#power.shortpersuasion[j] <- mean(c.T1vsC>0 & p.T1vsC < alpha/2)
power.shorttreatments[j] = mean(c.T1vsC>0 & c.T2vsC>0 & p.T1vsC
   < alpha/2 & p.T2vsC< alpha/2)
power.persuasion[j] = mean(c.T1vsC>0 & c.T3vsC>0 & p.T1vsC <
   alpha/2 & p.T3vsC < alpha/2)
power.empathy[j] = mean(c.T2vsC>0 & c.T4vsC>0 & p.T2vsC < alpha/
   2 & p.T4vsC < alpha/2)
power.persuasionM[j] = mean(c.T1MvsC>0 & p.T1MvsC<alpha/2)
power.empathyM[j] = mean(c.T2MvsC>0 & p.T2MvsC<alpha/2)
#power.shortpersuasionVempathy[j] = mean(c.T2vsT1>0 & p.T2vsT1 <
    alpha/2)
#power.persuasionVempathy[j] =mean(c.T2vsT1>0 & c.T4vsT3>0 & p.
   T2vsT1 < alpha/2 & p.T4vsT3 <alpha/2)
power.alltreatments[j] <- mean(c.T1vsC>0 & c.T2vsC>0 & c.T3vsC>0
    & c.T4vsC>0 & c.T1MvsC>0 & c.T2MvsC>0
                                 & p.T1vsC < alpha/2 & p.T2vsC <
                                    alpha/2 & p.T3vsC<alpha/2 & p.
                                    T4vsC<alpha/2 & p.T1MvsC<alpha
                                    /2 & p.T2MvsC<alpha/2)
#power.fullranking[j] <- mean(c.T1vsC>0 & c.T2vsC>0 & c.T3vsC>0
   & c.T4vsC>0 & c.T2vsT1 > 0 &c.T3vsT1>0 & c.T4vsT1>0 & c.
   T3vsT2>0 & c.T4vsT2>0 & c.T4vsT3>0 & p.T1vsC < alpha/2 & p.
   T2vsC<alpha/2 & p.T3vsC<alpha/2 & p.T4vsC<alpha/2& p.T2vsT1 <
    alpha/2 & p.T3vsT1 < alpha/2 & p.T4vsT1 < alpha/2 & p.T3vsT2
    < alpha/2 & p.T4vsT2 < alpha/2 & p.T4vsT3 < alpha/2) #diff
   btwn all treatments too
print(j)
```

```
}

plot(possible.ns, power.alltreatments30 , ylim=c(0,1),pch=16, ylab=
    "Power", xlab="n size required")
#points(possible.ns, power.persuasion , col="green")
#points(possible.ns, power.empathy, col="blue")
#points(possible.ns, power.persuasionM , col="darkgreen")
#points(possible.ns, power.empathyM, col="darkblue")
points(possible.ns, power.alltreatments35 , col="red",pch=16)
abline(a = 0.8,b=0,col=3)

abline(v=4300,col=1,lty=3)#black-sd=30
abline(v=5500,col=2,lty=3)#red-sd=35
## with sd=29, power for all treatments N=3900
## with sd=35, power for all treatments N=5500
power.alltreatments35<-power.alltreatments
## with sd=30, power for all treatments N=4300
power.alltreatments30<-power.alltreatments
```

References

Albertson, Bethany and Shana Gadarian. 2013. Who's Afraid of Immigration? The Effects of Pro- and Anti-Immigrant Threatening Ads among Latinos, African Americans and Whites. In *Immigration and Public Opinion in Liberal Democracies*, ed. Gary Freeman, Randall Hansen and David Leal. Routledge.

Albertson, Bethany and Shana Gadarian. 2015*a*. *Anxious politics: democratic citizenship in a threatening world*. New York, NY: Cambridge Univ Press.

Albertson, Bethany and Shana Gadarian. 2015*b*. "Ebola, anxiety, and public support for protective policies." *PS: Political Science and Politics* 48(01):8–9.

Braun, Henry. 1994. *The Collected Works of John W. Tukey*. 8th ed. ed. Chapman and Hall - CRC.

Broockman, David and Joshua Kalla. 2016. "Durably reducing transphobia: A field experiment on door-to-door canvassing." *Science* 352(6282):220–224.

Dancygier, Rafaela. 2010. *Immigration and conflict in Europe*. Cambridge University Press.

Facchini, Giovanni, Yotam Margalit and Hiroyuki Nakata. 2016. "Reducing Public Hostility to Immigration : The Impact of Information Campaigns.".

Hainmueller, Jens and Daniel J. Hopkins. 2014. "Public Attitudes Toward Immigration." *Annual Review of Political Science* 17(1):225–249.

Hainmueller, Jens and Daniel J. Hopkins. 2015. "The Hidden American Immigration Consensus: A Conjoint Analysis of Attitudes toward Immigrants." *American Journal of Political Science* 59(3):529–548.

Hainmueller, Jens, Daniel J. Hopkins and Teppei Yamamoto. 2014. "Causal inference in conjoint analysis: Understanding multidimensional choices via stated preference experiments." *Political Analysis* 22(1):1–30.

Kalkan, Kerem Ozan, Geoffrey C. Layman and Eric M. Uslaner. 2009. ""Bands of Others"? Attitudes toward Muslims in Contemporary American Society." *The Journal of Politics* 71(03):847.

Lin, Winston. 2013. "Agnostic notes on regression adjustments to experimental data: reexamining Freedman's critique." *The Annals of Applied Statistics* 7(1):295–318.

Neuliep, J.W. and McCroskey, J.C. 1997. "Development of a US and generalized ethnocentrism scale." *Communication Research Reports* 14:385–398.

Panagopoulos, Costas. 2006. "The polls-trends: Arab and Muslim Americans and Islam in the aftermath of 9/11." *Public Opinion Quarterly* 70(4):608–624.

Savelkoul, Michael, Peer Scheepers, Jochem Tolsma and Louk Hagendoorn. 2011. "Anti-Muslim attitudes in the Netherlands: Tests of contradictory hypotheses derived from ethnic competition theory and intergroup contact theory." *European Sociological Review* 27(6):741–758.

Sniderman, Paul M, Louk Hagendoorn and Markus Prior. 2004. "Predisposing Factors and Situational Triggers: Exclusionary Reactions to Immigrant Minorities." *The American Political Science Review* 98(1):35–49.
