# Supplementary material for: Americans preferred Syrian refugees who are female, English-speaking, and Christian on the eve of Donald Trump’s election
Source: PLoS One. 2019 Oct 10;14(10):e0222504. doi: 10.1371/journal.pone.0222504 (PMC6786519; doi:10.1371/journal.pone.0222504)
Supplement: S2 File — (PDF) [file pone.0222504.s002.pdf]

Sampling Information

This study was part of a larger survey experiment on 5,400 American adult citizens. We rely here on a random sub-sample of 1,800 respondents. YouGov provided a representative sample of American citizens matched on gender, age, race, education, party identification, ideology, and political interest with the 2010 American Community Survey, the November 2010 Current Population Survey, and the 2007 Pew Religious Life Survey. The sampling frame was constructed by stratified sampling from the full 2010 American Community Survey (ACS) sample with selection within strata by weighted sampling with replacements (using the person weights on the public use file). Data on voter registration status and turnout were matched to this frame using the November 2010 Current Population Survey. Data on interest in politics and party identification were then matched to this frame from the 2007 Pew Religious Life Survey. The matched cases were weighted to the sampling frame using propensity scores. The matched cases and the frame were combined and a logistic regression was estimated for inclusion in the frame. The propensity score function included age, gender, race/ethnicity, years of education, voter registration, ideology, non-identification with a major party, and census region. The propensity scores were grouped into deciles of the estimated propensity score in the frame and post-stratified according to these deciles. The larger experiment sample size of 5,400 was then combined, and combined weights were post-stratified to match a full stratification of four category age, four category race, gender, and four category education.
