# Supplementary figures and images for: Americans preferred Syrian refugees who are female, English-speaking, and Christian on the eve of Donald Trump’s election
Source: PLoS One. 2019 Oct 10;14(10):e0222504. doi: 10.1371/journal.pone.0222504 (PMC6786519; doi:10.1371/journal.pone.0222504)

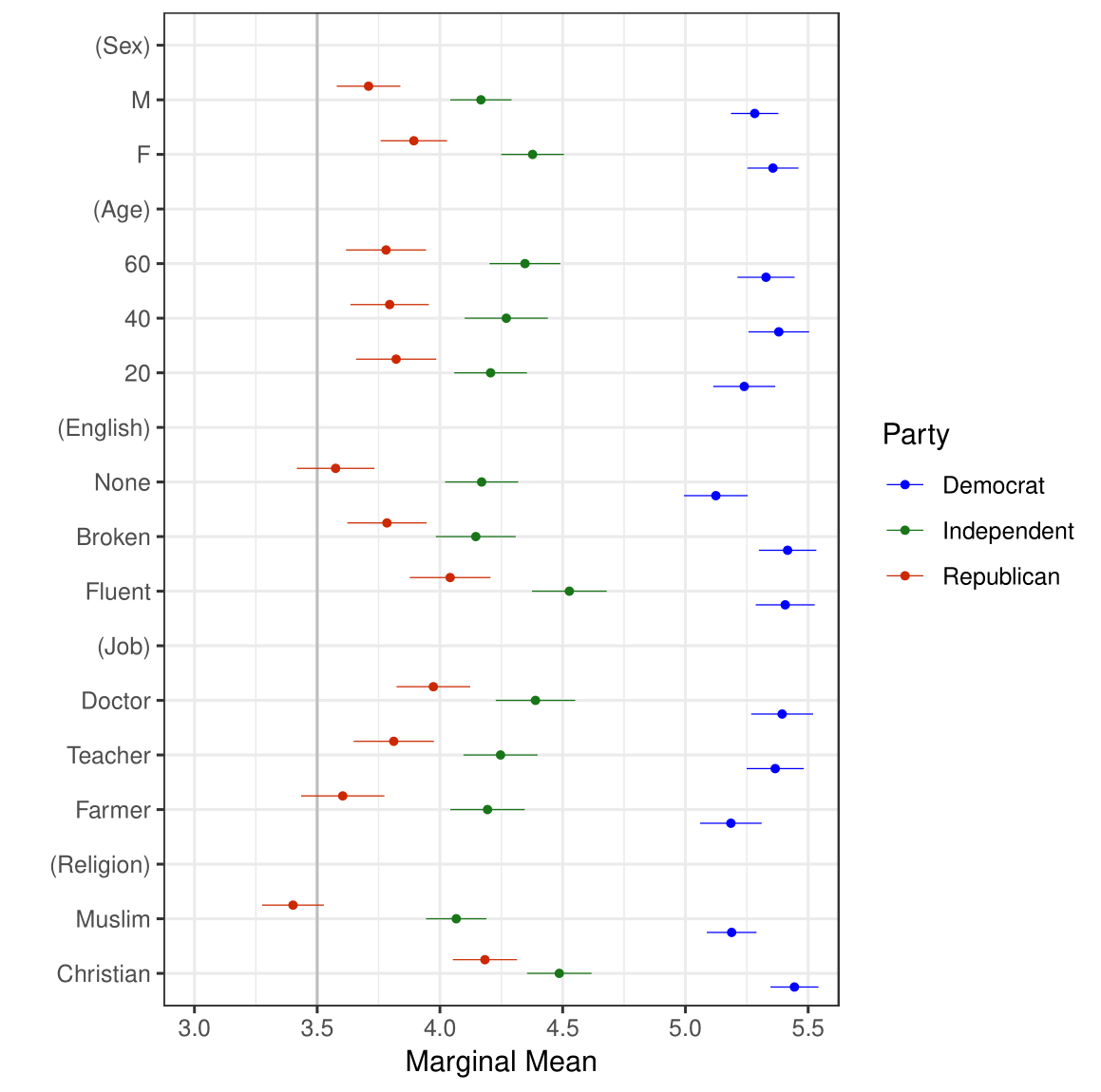

Supplement: S1 Fig — Categories are Democrat, Independent, and Republican. Confidence intervals are at 95%. (TIFF) [file pone.0222504.s003.tiff]

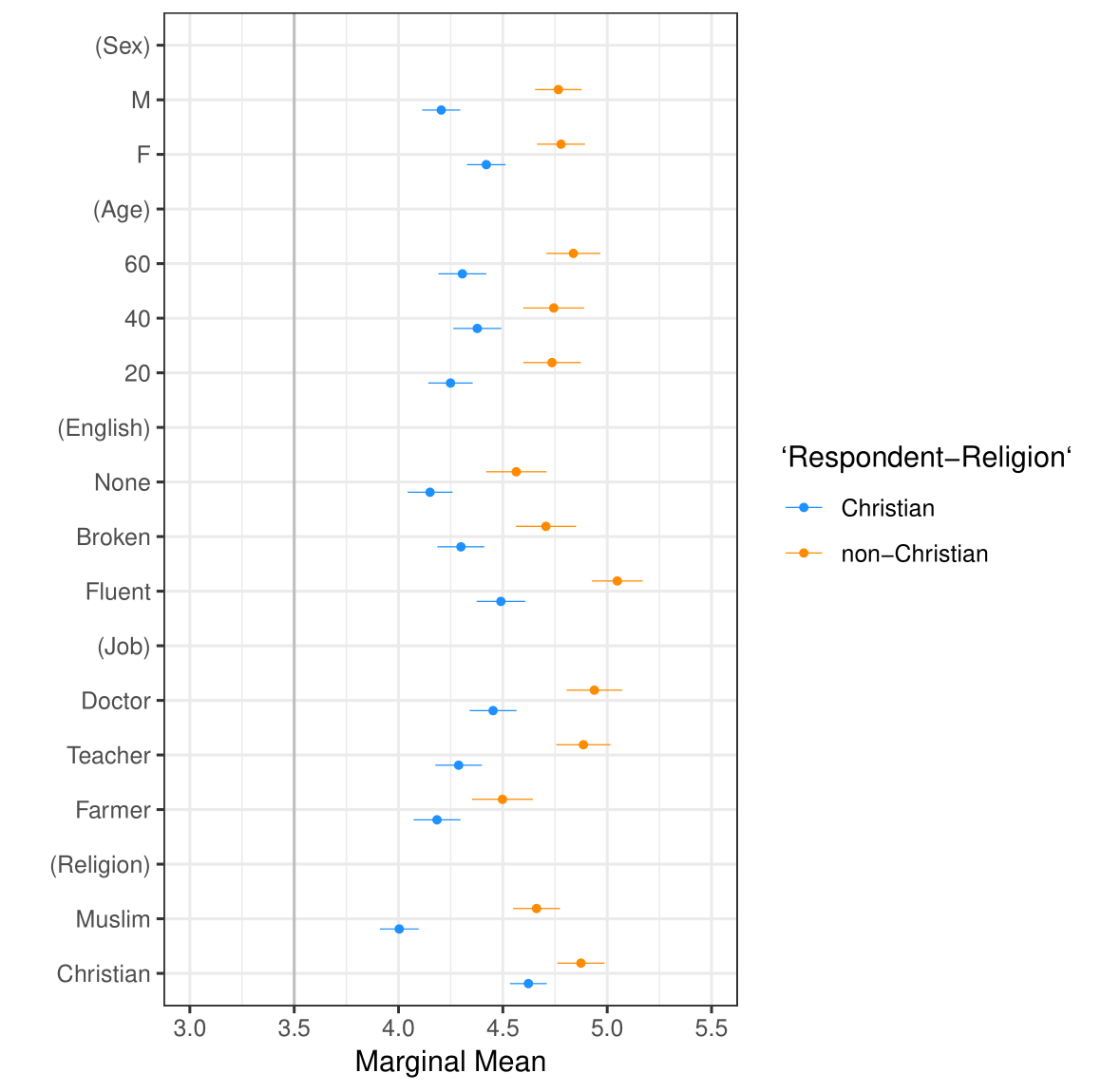

Supplement: S2 Fig — Categories are Christian and non-Christian. Confidence intervals are at 95%. (TIFF) [file pone.0222504.s004.tiff]

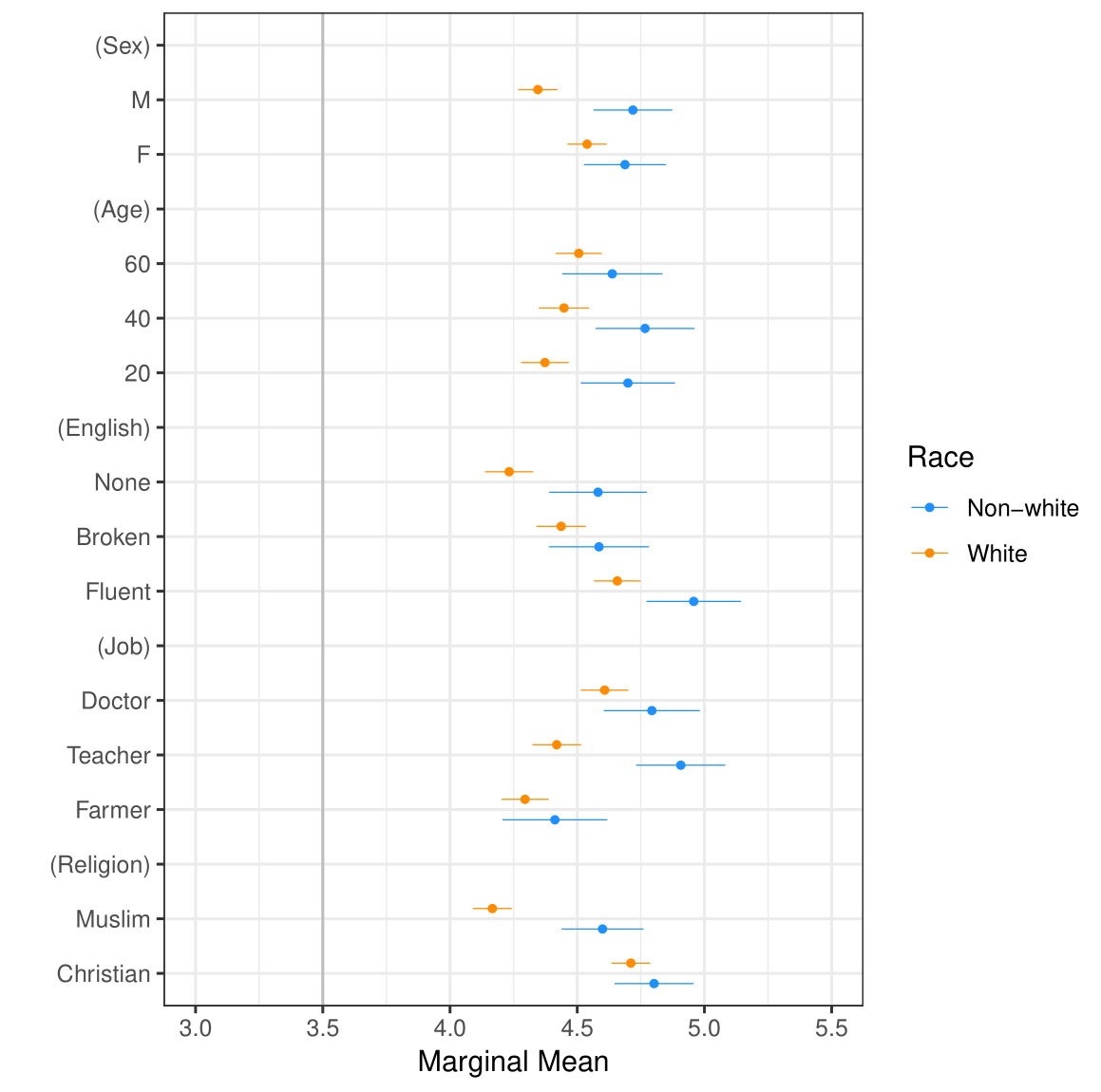

Supplement: S3 Fig — Categories include White and non-White respondents. Confidence intervals are at 95%. (TIFF) [file pone.0222504.s005.tiff]

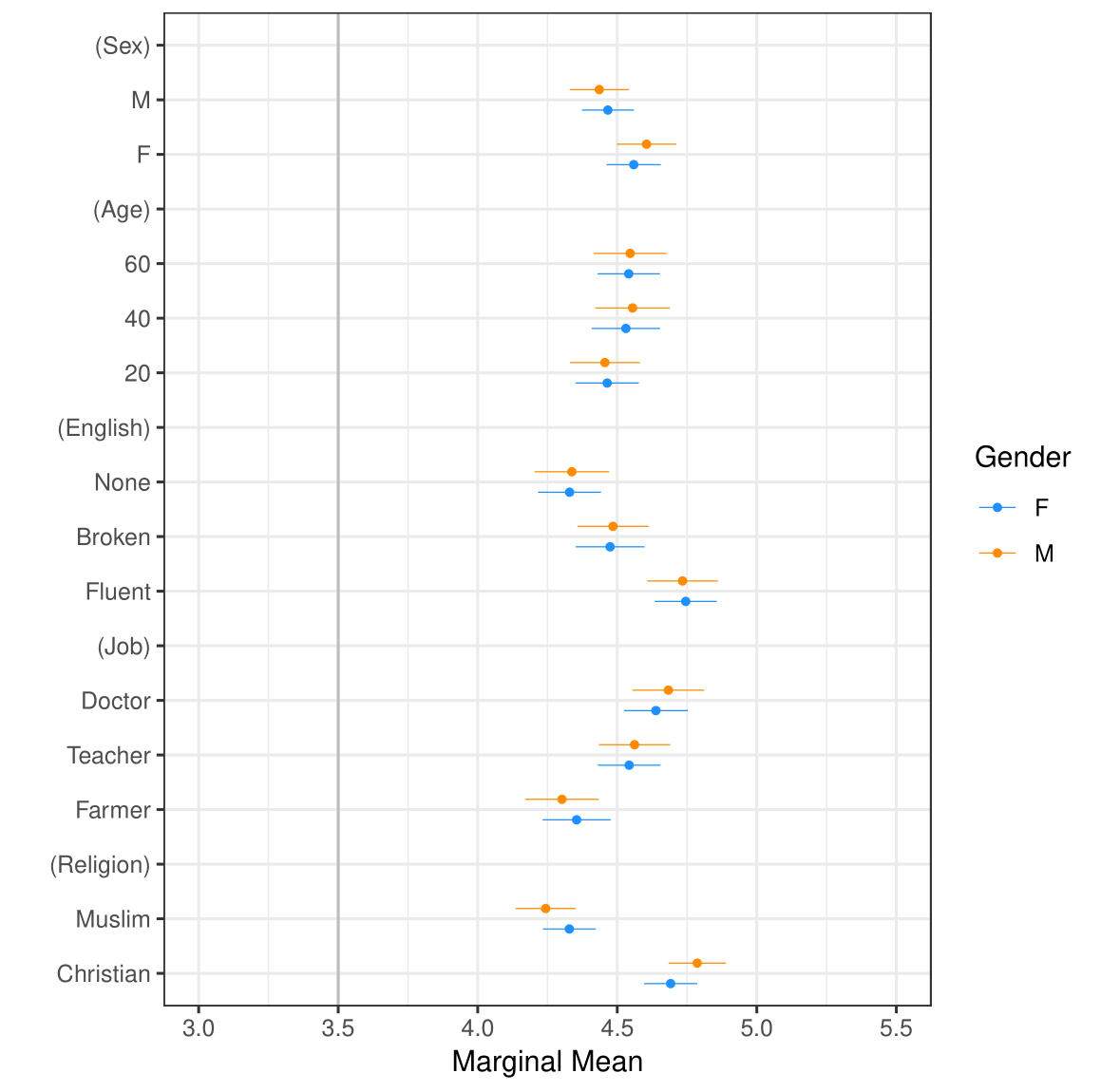

Supplement: S4 Fig — “F” are female respondents and “M” indicate male respondents. Confidence intervals are at 95%. (TIFF) [file pone.0222504.s006.tiff]

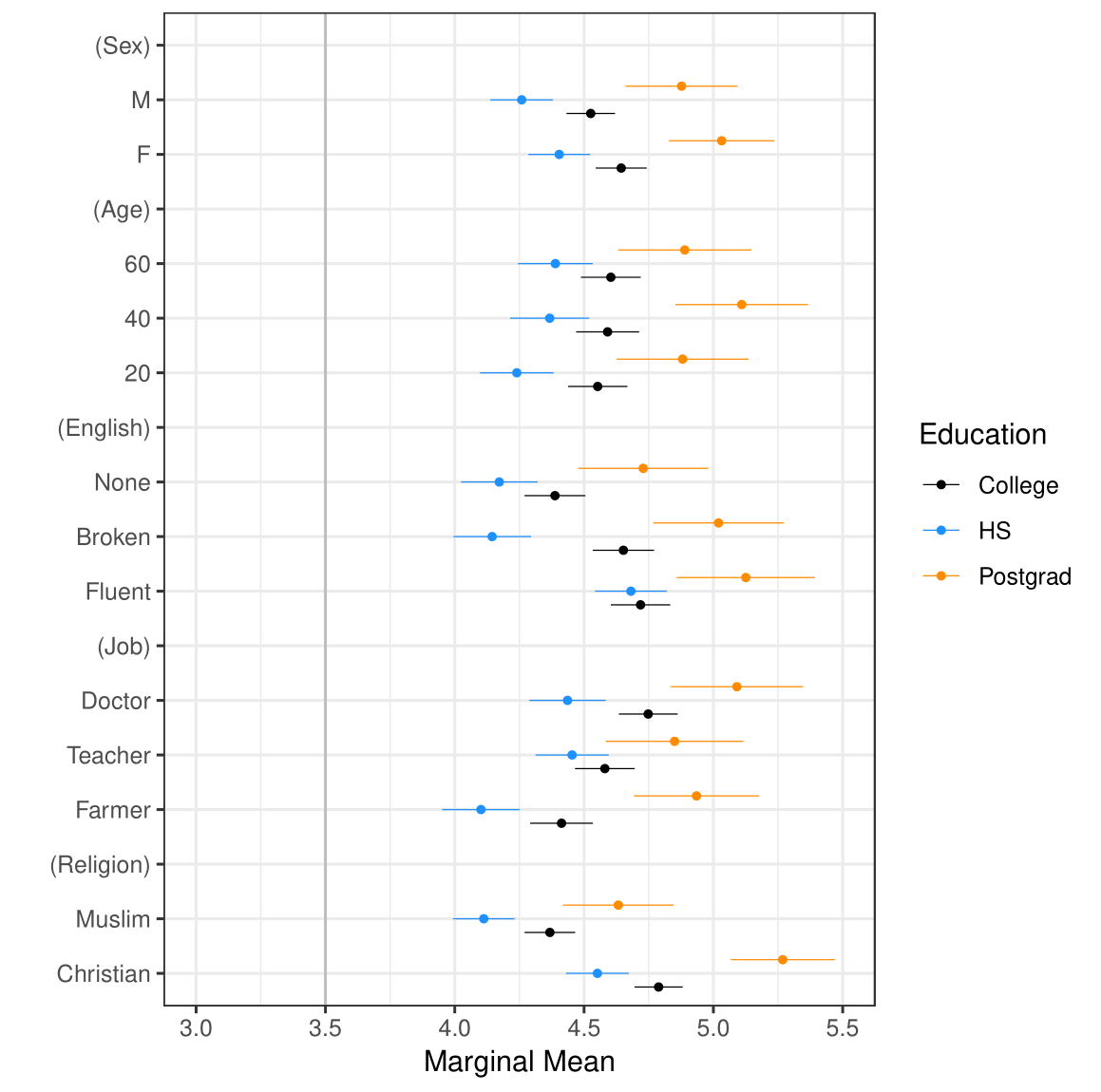

Supplement: S5 Fig — Education categories are respondents who have high school, college, and post-graduate degrees. Confidence intervals are at 95%. (TIFF) [file pone.0222504.s007.tiff]

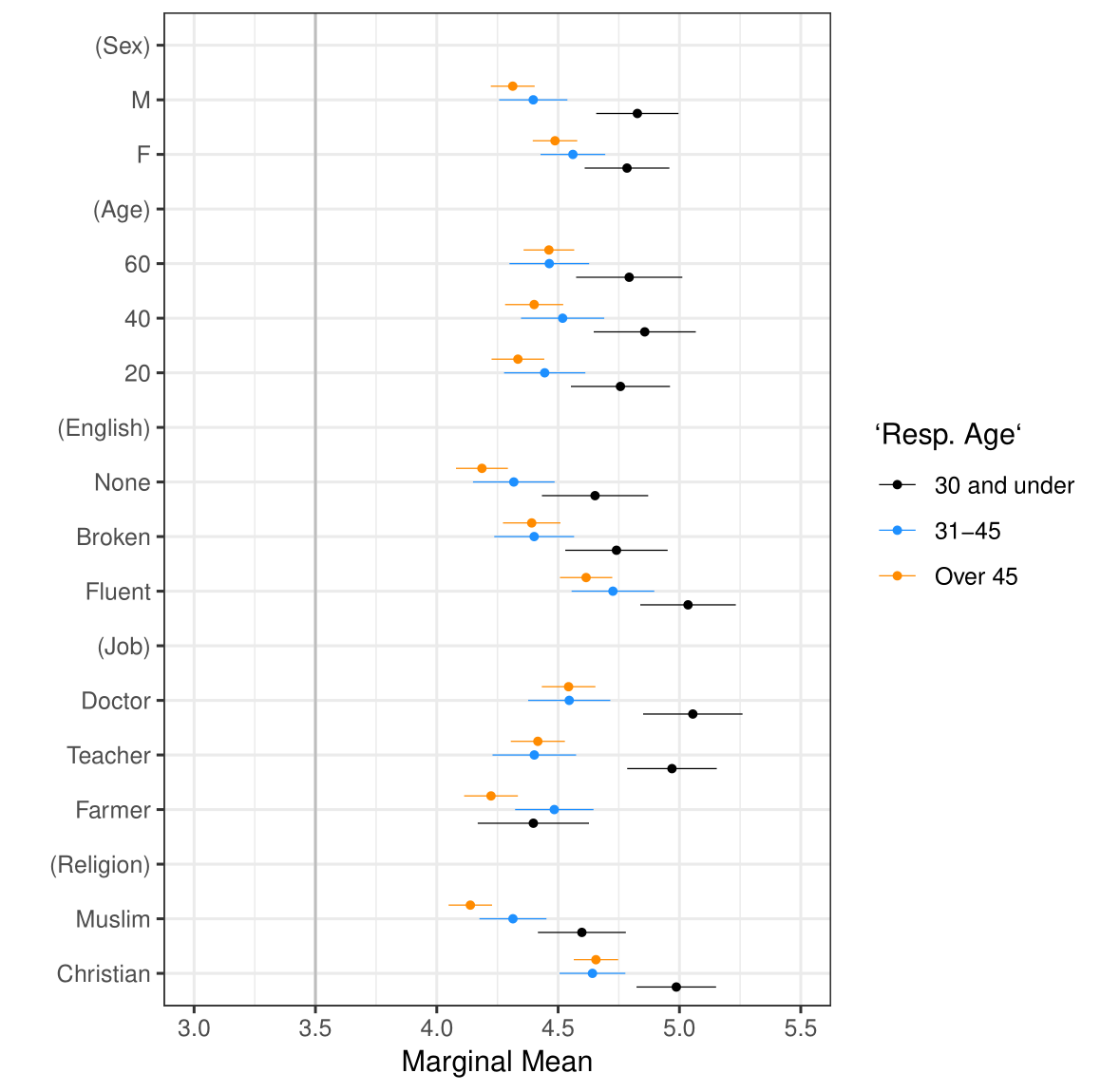

Supplement: S6 Fig — Categories are age 30 and under, ages 31-45, and over 45. Confidence intervals are at 95%. (TIFF) [file pone.0222504.s008.tiff]

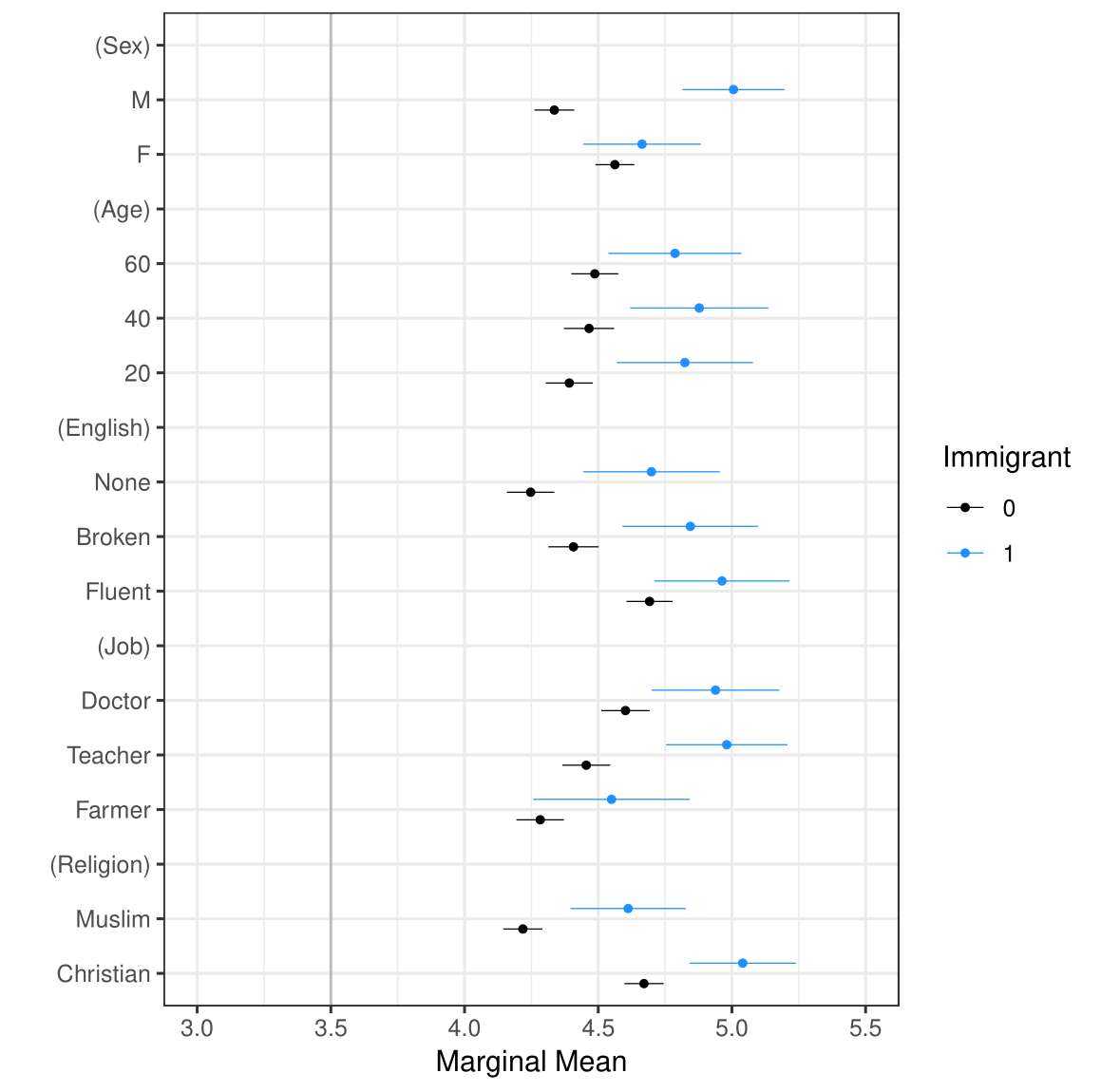

Supplement: S7 Fig — Group 1 are respondents who are immigrants to the U.S. or have at least one parent who is an immigrant. Group 0 are respondents who are not immigrants, nor have parents who are immigrants, and either have no immigrants in their grandparents’ generation or at least one immigrant in their grandparents’ generation. (TIFF) [file pone.0222504.s009.tiff]

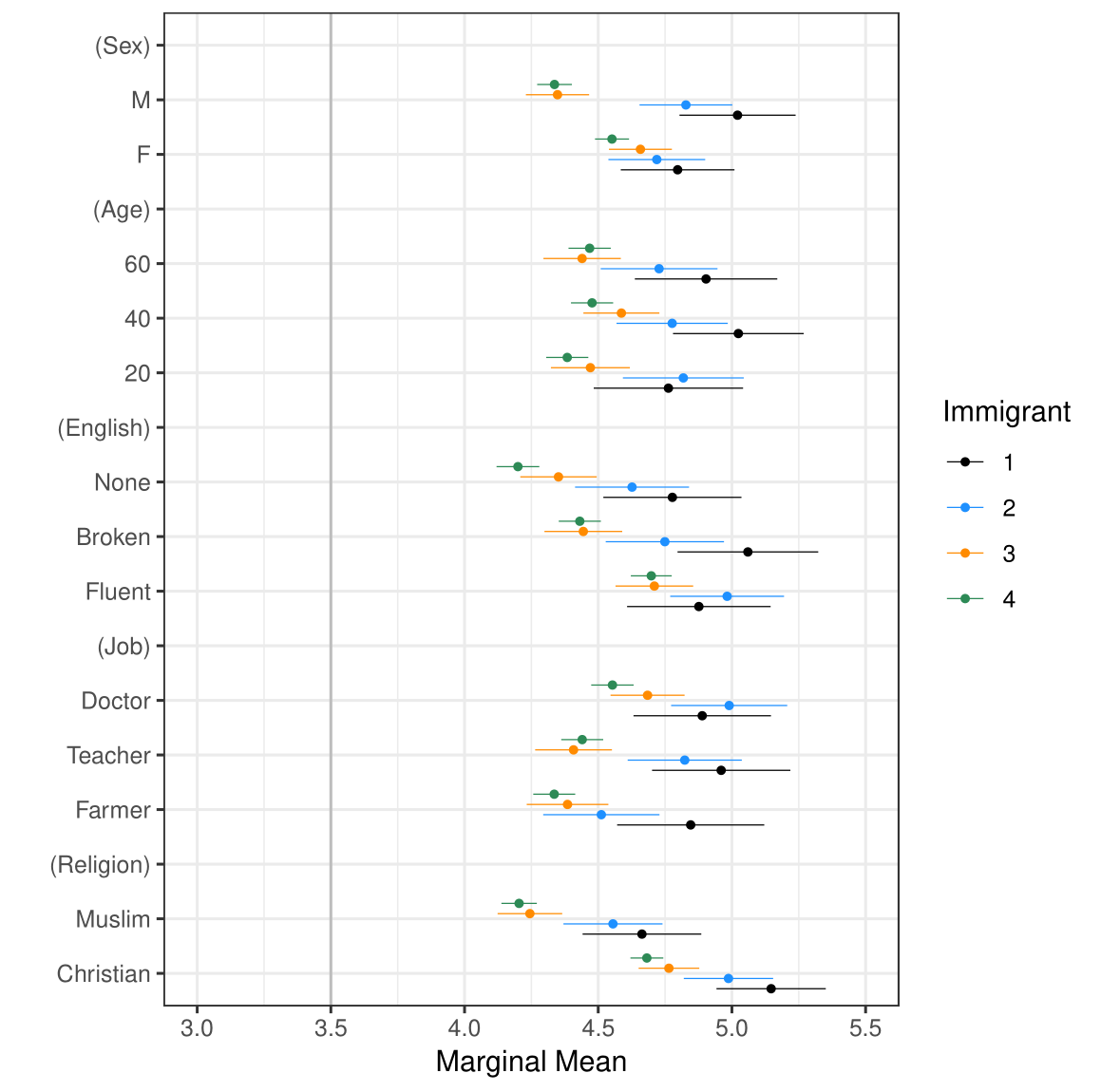

Supplement: S8 Fig — Group 1 are respondents who are immigrants to the U.S., Group 2 respondents have at least one parent who is an immigrant, Group 3 has at least one grandparent who is an immigrant, and Group 4 has no immigrant history within their own, their parents’ and their grandparents’ generations. (TIFF) [file pone.0222504.s010.tiff]

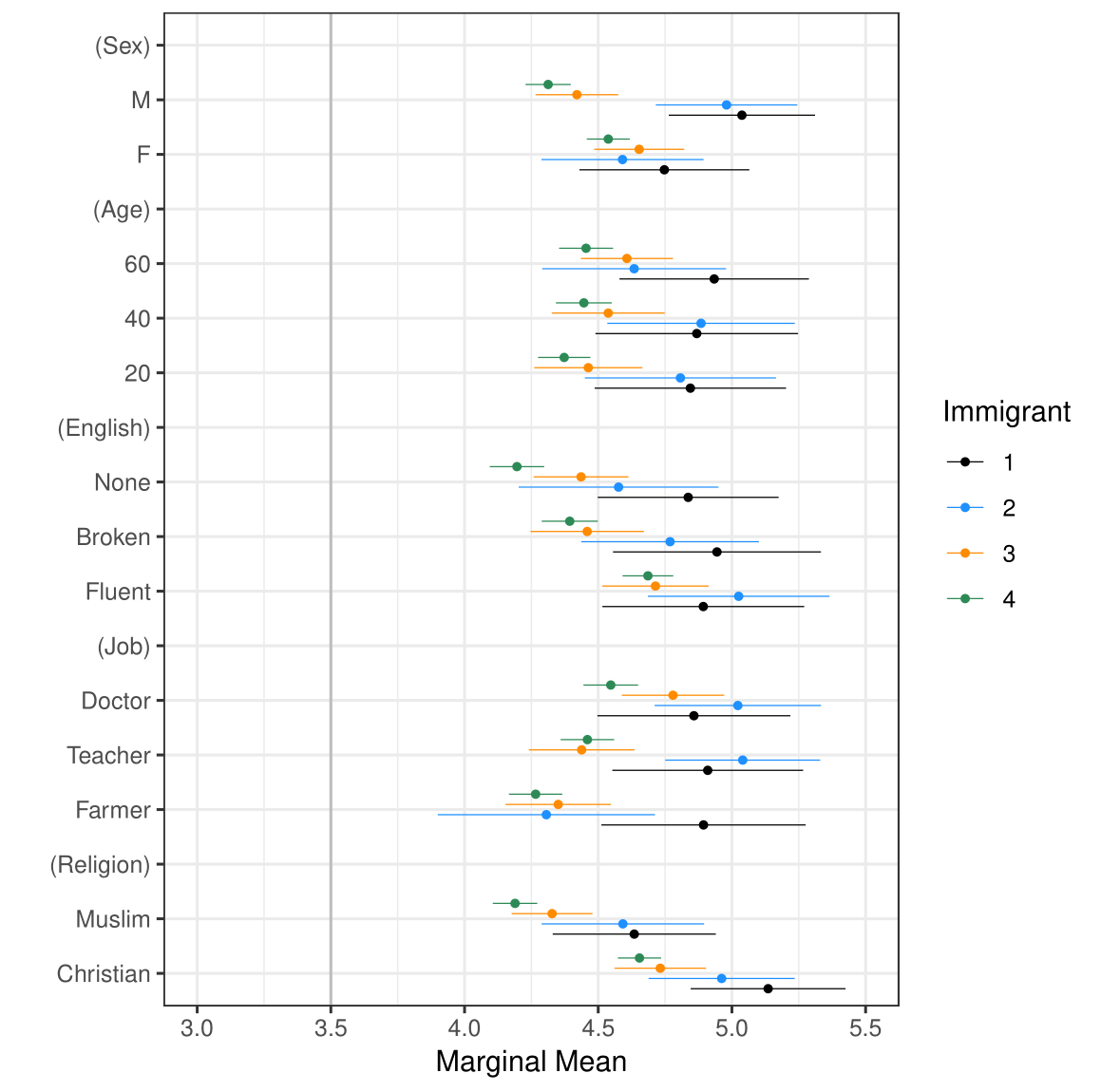

Supplement: S9 Fig — Group 1 are respondents who are immigrants to the U.S., Group 2 respondents have at least one parent who is an immigrant, Group 3 has at least one grandparent who is an immigrant, and Group 4 has no immigrant history within their own, their parents’ and their grandparents’ generations. (TIFF) [file pone.0222504.s011.tiff]
